# Web-Based Interventions Targeting Cardiovascular Risk Factors in Middle-Aged and Older People: A Systematic Review and Meta-Analysis

**DOI:** 10.2196/jmir.5218

**Published:** 2016-03-11

**Authors:** Cathrien RL Beishuizen, Blossom CM Stephan, Willem A van Gool, Carol Brayne, Ron JG Peters, Sandrine Andrieu, Miia Kivipelto, Hilkka Soininen, Wim B Busschers, Eric P Moll van Charante, Edo Richard

**Affiliations:** ^1^ Academic Medical Center Department of Neurology University of Amsterdam Amsterdam Netherlands; ^2^ Institute of Health and Society Newcastle University Institute for Ageing Newcastle University Newcastle upon Tyne United Kingdom; ^3^ Cambridge Institute of Public Health Department of Public Health and Primary Care University of Cambridge Cambridge United Kingdom; ^4^ Academic Medical Center Department of Cardiology University of Amsterdam Amsterdam Netherlands; ^5^ Inserm U1027 Department of Epidemiology and Public Health Toulouse University Hospital Toulouse France; ^6^ Aging Research Center Alzheimer Disease Research Center Karolinska Institutet Stockholm Sweden; ^7^ Department of Neurology University of Eastern Finland and Kuopio University Hospital Kuopio Finland; ^8^ Academic Medical Center Department of General Practice University of Amsterdam Amsterdam Netherlands; ^9^ Department of Neurology Radboud University Medical Center Nijmegen Netherlands

**Keywords:** eHealth, cardiovascular disease, prevention, older people, aging, systematic review, meta-analysis

## Abstract

**Background:**

Web-based interventions can improve single cardiovascular risk factors in adult populations. In view of global aging and the associated increasing burden of cardiovascular disease, older people form an important target population as well.

**Objective:**

In this systematic review and meta-analysis, we evaluated whether Web-based interventions for cardiovascular risk factor management reduce the risk of cardiovascular disease in older people.

**Methods:**

Embase, Medline, Cochrane and CINAHL were systematically searched from January 1995 to November 2014. Search terms included cardiovascular risk factors and diseases (specified), Web-based interventions (and synonyms) and randomized controlled trial. Two authors independently performed study selection, data-extraction and risk of bias assessment. In a meta-analysis, outcomes regarding treatment effects on cardiovascular risk factors (blood pressure, glycated hemoglobin A1c (HbA1C), low-density lipoprotein (LDL) cholesterol, smoking status, weight and physical inactivity) and incident cardiovascular disease were pooled with random effects models.

**Results:**

A total of 57 studies (N=19,862) fulfilled eligibility criteria and 47 studies contributed to the meta-analysis. A significant reduction in systolic blood pressure (mean difference –2.66 mmHg, 95% CI –3.81 to –1.52), diastolic blood pressure (mean difference –1.26 mmHg, 95% CI –1.92 to –0.60), HbA1c level (mean difference –0.13%, 95% CI –0.22 to –0.05), LDL cholesterol level (mean difference –2.18 mg/dL, 95% CI –3.96 to –0.41), weight (mean difference –1.34 kg, 95% CI –1.91 to –0.77), and an increase of physical activity (standardized mean difference 0.25, 95% CI 0.10-0.39) in the Web-based intervention group was found. The observed effects were more pronounced in studies with short (<12 months) follow-up and studies that combined the Internet application with human support (blended care). No difference in incident cardiovascular disease was found between groups (6 studies).

**Conclusions:**

Web-based interventions have the potential to improve the cardiovascular risk profile of older people, but the effects are modest and decline with time. Currently, there is insufficient evidence for an effect on incident cardiovascular disease. A focus on long-term effects, clinical endpoints, and strategies to increase sustainability of treatment effects is recommended for future studies.

## Introduction

The field of eHealth is expanding the potential of contemporary medicine [1]. Global aging and its associated burden of cardiovascular disease may expand the scope for innovative Internet interventions [2,3]. Current cardiovascular risk management programs in primary care will become too expensive and, although they are highly effective in research settings [4-6], their effectiveness is markedly lower in daily life [7]. This evidence-practice gap has several causes [8]. Adherence to life-long lifestyle and medication regimens is a serious challenge, illustrated by long-term adherence rates in chronic diseases that average as low as 50% [9,10]. Web-based interventions are cheap, have a wide reach, and they enable self-management [11]. This renders Web-based interventions potentially powerful and scalable tools to enhance sustained adherence in cardiovascular risk management [12].

Older people form an important target population because cardiovascular risk reduction appears effective until old age [13-16]. In 2012, 42% of European people aged between 55 and 74 years used the Internet and this number is increasing [17]. Meta-analyses showed that Web-based interventions targeting single cardiovascular risk factors can induce improvements in adult populations [18-21]. However, optimal cardiovascular prevention and risk management practice, as affirmed by the European Society of Cardiology [22] and the American Heart Association [23], requires targeting the complete cardiovascular risk profile. This is particularly applicable for older people, who often have multiple risk factors or already suffered a cardiovascular event. A comprehensive approach would increase the value of Web-based interventions for daily practice. Currently, little is known about the effectiveness of Web-based interventions in older people.

In this systematic review and meta-analysis, we aim to answer the question whether Web-based interventions for cardiovascular risk factor management reduce cardiovascular risk and disease in older people.

## Methods

### Search Strategy and Selection of Eligible Studies

We performed a systematic literature search for randomized controlled trials (RCT) on Web-based interventions in older people targeting one or more cardiovascular risk factors and/or disease. Methods were predefined in a research protocol using the PRISMA checklist and the Systematic Reviews Guidelines of the Center of Reviews and Dissemination (Multimedia Appendix 1). We defined Web-based interventions as Web-based participant-centered treatment or prevention programs delivered via the Internet and interacting with the participant in a tailored fashion [24,25]. Internet had to be the main medium through which the intervention was delivered, but other media (phone, face-to-face) could be included too. We excluded the following eHealth interventions: telemonitoring, telemedicine, and mobile phone-mediated interventions. The target of the intervention had to be one or more cardiovascular risk factors and/or cardiovascular disease. Thus, we included interventions for both primary and secondary prevention of cardiovascular disease [22]. The target population had to have a mean age of 50 years or older and could have a mixed level of cardiovascular risk (one or more cardiovascular risk factors or established cardiovascular disease).

Main outcomes of interest were incident cardiovascular disease (myocardial infarction, angina pectoris, heart failure, stroke or transient ischemic attack, and peripheral arterial disease), cardiovascular mortality and overall mortality, and changes in cardiovascular risk factors including blood pressure (BP), glycated hemoglobin A_1c_ (HbA_1c_), low-density lipoprotein (LDL) cholesterol, smoking status, weight, level of physical exercise, or a composite cardiovascular risk score.

We performed a comprehensive literature search in the EMBASE, Medline, CINAHL, and Cochrane databases from 1995 onward (because the Internet was not widely available before then). Key search terms were cardiovascular risk factors and diseases (separate diseases and risk factors specified), terms related to aspects of cardiovascular risk management (eg, diet, exercise, BP control), Web-based interventions (including all definitions and synonyms), and RCT/review/meta-analysis. The search was last updated on November 3, 2014 by CRB. The comprehensive search strategy is provided in Multimedia Appendix 2. Studies were included if (1) they were on Web-based interventions targeting cardiovascular risk factors and/or disease, (2) study design was a RCT, (3) at least 50 patients were included, (4) mean age was at least 50 years, (5) the duration of the intervention was 4 or more weeks and follow-up was 3 or more months, (6) at least one of the outcomes of our interest was reported, and (7) language was English. Study selection was performed by two independent researchers (CRB and BS) by means of screening of titles and abstracts, and thereafter reading full texts on the basis of the inclusion criteria. If two publications described the same trial, the paper that reported the primary outcomes of the trial was included. Disagreements were resolved by discussion or by a third investigator (ER). We assessed reviews and meta-analyses encountered with our search strategy to check for additional relevant articles.

### Data Extraction

Two reviewers (BS and CRB) extracted data using a predefined data extraction form (Multimedia Appendix 3) for half of the included articles and checked each other’s results. Extracted information included study characteristics, patient baseline characteristics, characteristics of the intervention and control conditions, and available data on clinical and intermediate outcomes. For BP, glucose control, weight, lipids, and physical activity level, we extracted all baseline and follow-up levels, change scores or mean differences. Corresponding authors were contacted if needed. We used an adapted Cochrane Risk of Bias Tool to evaluate randomization procedures, representativeness of study populations, blinding of outcome assessors (blinding of participants was usually not possible due to study design), completeness of outcome data, and completeness of reporting.

### Meta-Analysis

For categorical variables, we calculated odds ratios with 95% confidence intervals. We estimated pooled odds ratios with Mantel-Haenszel random-effects models. For continuous outcomes, mean differences or standardized mean differences (Hedges’ *g* effect sizes) with 95% confidence intervals were calculated. We estimated pooled effects with DerSimonian and Laird random-effects models. All HbA_1c_ values were converted to percentages. All LDL cholesterol values were converted to mg/dL. All weight values were converted to kg. For level of physical activity, which was assessed with various instruments, we calculated standardized mean differences and 95% confidence intervals. If mean differences or standardized mean differences were reported, we included them directly in the pooled analyses. If not, we calculated change scores (difference between baseline and follow-up within group) or values assessed at follow-up. If values were measured at multiple time points, we used the values recorded at the last follow-up contact.

For studies with multiple arms, we included only one intervention arm in the meta-analysis in order not to create “unit-of-analysis” error by double counting the control group. Where possible, we selected the Internet-only intervention arm. No data were imputed.

We estimated pooled effects for all single cardiovascular risk factors. To address the overall question of efficacy of Web-based interventions for cardiovascular risk factor management, we evaluated the effect on cardiovascular composite scores, clinical outcomes (cardiovascular morbidity and mortality), and pooled the standardized primary outcomes of all studies. We used the primary outcomes as defined by the authors of the studies.

Funnel plots were inspected to assess for potential publication bias. Statistical heterogeneity was assessed using Q and I^2^ tests. We explored reasons for heterogeneity by jackknife analysis and subgroup analyses. We assessed the following factors in subgroup analyses: study duration (predefined, short term [<12 months] versus long term [≥12 months]), type of cardiovascular prevention (primary versus secondary) [22], and type of intervention (Internet only or “blended” [Internet application combined with human support]). Subgroup analyses were performed on the studies used for the analysis on primary outcomes only. The latter subgroup analysis (on type of intervention) consisted of two separate analyses, one to evaluate the Internet-only interventions versus the control conditions and one to evaluate the blended interventions versus control conditions. In case a study tested both types of interventions with a multiple-arm design, the appropriate arm was included for each analysis. In addition, we performed a mixed effects meta-regression using the unrestricted maximum likelihood method to explore the association between study duration and effect size (standardized primary outcome). Last, we performed sensitivity analyses for the different domains of the risk-of-bias assessment by repeating the analysis on standardized primary outcomes in subgroups of studies with low risk of bias versus studies with an unclear or high risk of bias. For this analysis, we wanted to include all studies that contributed to one of the meta-analyses. Therefore, we complemented the sample of studies with defined primary outcomes that were cardiovascular risk factors of interest with studies that had not defined their primary outcome. If there was no defined primary outcome, we used the cardiovascular risk factor that was targeted most directly in the intervention studied. We used Review Manager 5.2 to draw the risk-of-bias assessment figure and to calculate standard deviations or 95% confidence intervals in cases where only standard errors were available in the original data. We used Microsoft Office Excel version 10, SPSS version 20, and Comprehensive Meta Analysis version 2.2.064 for the statistical analyses.

## Results

### Study Selection

**Figure 1:**
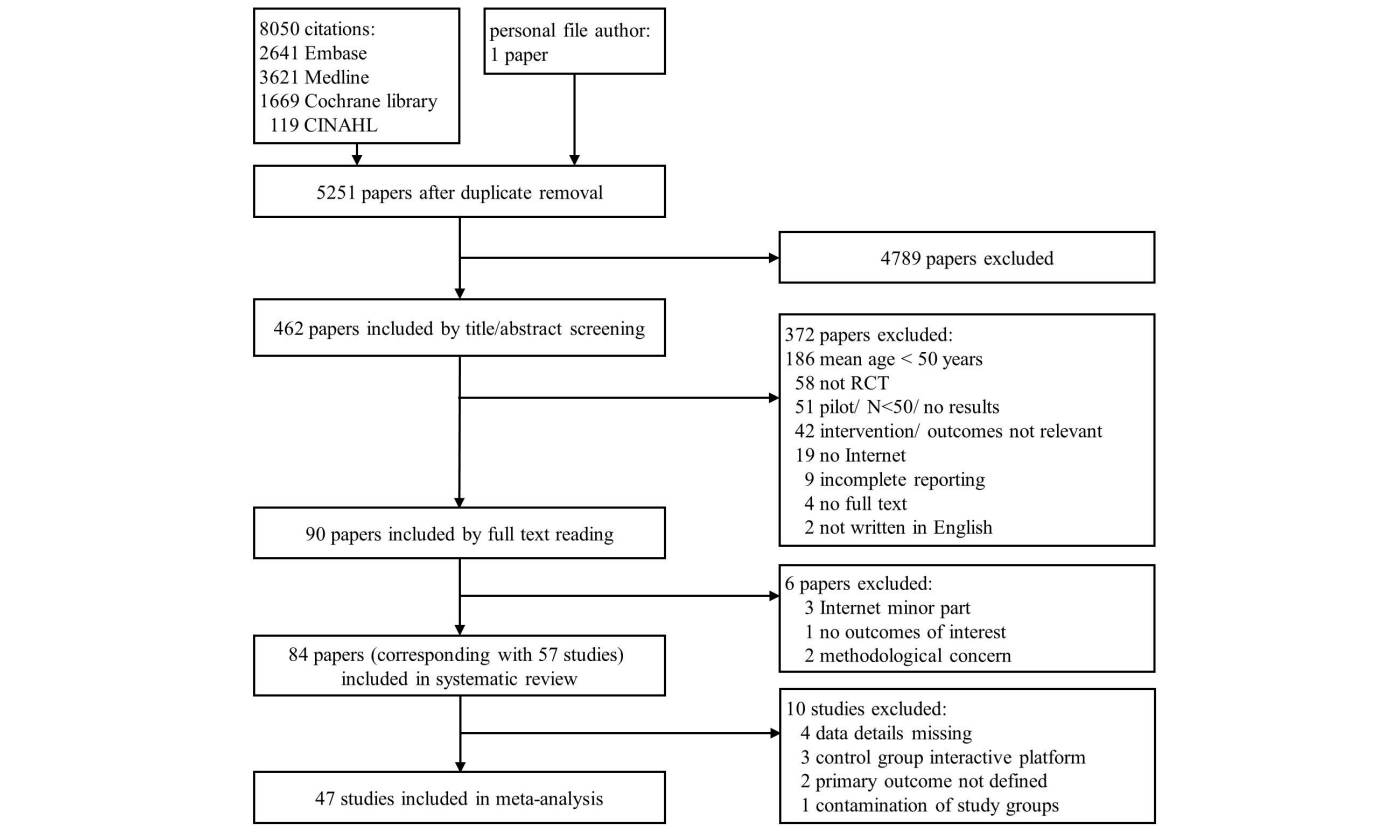
Prisma flowchart illustrating literature search.

The search yielded 5251 papers after removal of duplicates. We did not identify additional studies by searching reference lists. After screening of titles and abstracts, 462 papers remained. Review of these full texts resulted in 57 RCTs (corresponding with 84 papers) that fulfilled the selection criteria and were included in the systematic review. We contacted 16 authors to request additional data: nine authors responded and three authors complied with our request. Out of this final selection, 47 studies could be included in the meta-analysis (see Figure 1 for PRISMA flowchart).

### Study Characteristics

The 57 RCTs included 19,862 individuals (Tables 1-5). Study sample size ranged from 61 to 2140 participants. Median study duration was 9 months (interquartile range [IQR] 6, range 3-60 months). The mean dropout rate was 15% (range 0%-62%). The mean age of the study populations ranged from 50 to 71 years. In only 7 studies were all participants older than 50 years of age. All participants had an increased risk of cardiovascular disease: 46 studies conducted primary prevention (control of cardiovascular risk factors or diabetes) and 11 studies conducted secondary prevention. In 41 studies, the intervention targeted a single cardiovascular risk factor; in 16 studies, multiple risk factors were addressed. We found no studies on interventions for smoking cessation meeting our inclusion criteria. In most studies, the primary outcome was change in a specific cardiovascular risk factor targeted by the intervention. Sixteen studies reported on clinical outcomes including new cardiovascular events [26-31] and mortality rates [29-41] as a part of adverse event monitoring. All interventions included lifestyle education and were participant-centered. Forty-four studies stimulated self-management by means of goal setting and self-monitoring. Half of interventions were stand-alone Internet platforms and the other half were “blended” (ie, the platforms were supported by a nurse or another health care professional). Intervention usage was reported by 22 studies. The median percentage of participants logging in to the intervention platform was 72% (range 33%-100%).

### Quality Assessment

Methodological quality of the included studies varied (Multimedia Appendix 4). Most studies adequately described the randomization and allocation concealment procedures. Due to the nature of the interventions, none of the studies had a double-blind design. In 20 studies, outcome assessors were blinded [27,29,30,32,34-40,42,52,54,57,59,61,64,67,81], in 19 studies blinding was not mentioned or unclear [28,31,33,41,43,44,46,48,53,56,60,63,65,69,71,74,76,77,80], and in 18 studies outcome assessors were not blinded [26,45,47,49-51,55,58,62,66,68,70,72,73,75,78,79,82].

### Effect of Web-Based Interventions on Single Risk Factors

Of the 57 studies included in the systemic review, 47 studies [26-32,34-42,44-53,55-60,62,64-68,70,73,74,76-79,81,82] provided sufficient information to be included in the meta-analysis. The mean age of the study populations of these 46 studies had the same range as the complete sample of 57 studies.

#### Systolic and Diastolic Blood Pressure

**Figure 2:**
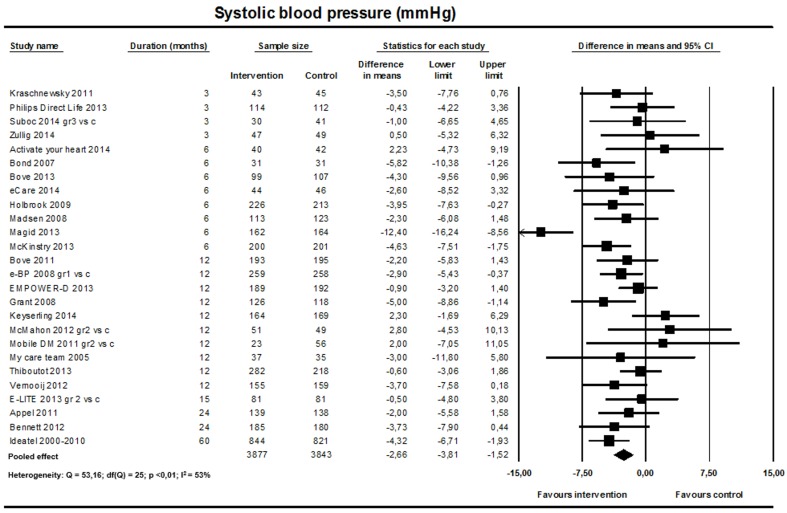
Effect on systolic blood pressure (26 studies).

**Figure 3:**
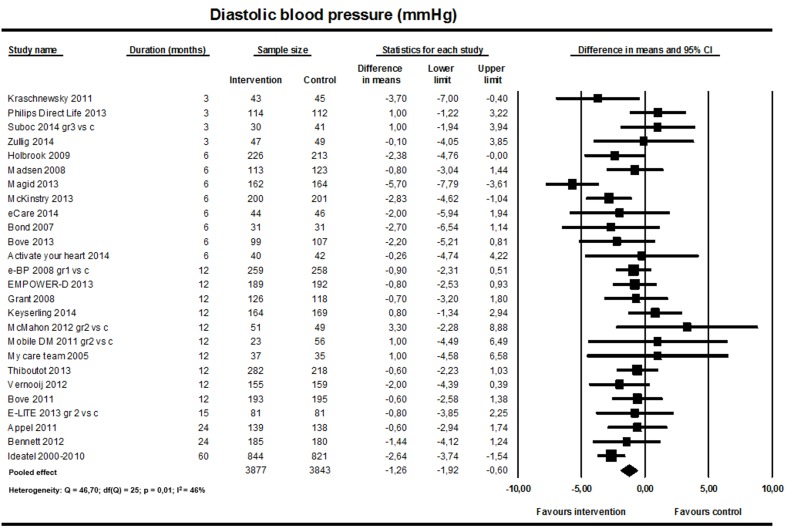
Effect on diastolic blood pressure (26 studies).

The pooled analysis showed a significant reduction in both systolic and diastolic BP favoring the intervention (26 studies; n=7720; Figures 2 and 3). For systolic BP, the weighted mean difference was –2.66 mmHg (95% CI –3.81 to –1.52; I^2^=53%). For diastolic BP, the weighted mean difference was –1.26 mmHg (95% CI –1.92 to –0.60; I^2^=46%).

**Table 1:** Characteristics of the studies included for the systematic review: interventions targeting diabetes.^a^

Study	Setting and study length	Participants	Age (years), mean (SD)	Sex (% female)	Intervention	Control	Primary; secondary outcomes
Bond 2010 [42]	2-arm RCT; USA; 6 m	62 people with DM via university/veteran clinic	67.2 (6.0)	45	Website: education, self-monitoring (glucose, exercise, weight, BP, medication), forum; nurse support (email, chat)	Standard diabetes care	HbA_1c_, BP, weight, total cholesterol, HDL cholesterol
IDEATEL 2000-2010 [34]	2-arm RCT; USA; 60 m	1665 Medicare recipients with DM	70.9 (6.7)	63	Online home telemedicine unit: nurse support (video chat), Web portal for self-monitoring (glucose, BP), education	Standard diabetes care	HbA_1c_, systolic BP, diastolic BP, total cholesterol, LDL cholesterol
D-net 2001 [43]	4-arm RCT; USA; 10 m	320 people with DM2, Internet, from 16 GPs	59 (9.2)	53	Website: (1) Self-management (glucose), coach support; (2) education, forum; (3) 1 and 2 combined^b^	(4) Information on medical and lifestyle aspects of diabetes	Not defined; behavioral, biological, and psychosocial outcomes
My path 2010 [44]	3-arm RCT; USA; 12 m	463 Medicare recipients with DM2, BMI ≥25 kg/m^2^ or ≥1 CV risk factor, Internet	58.4 (9.2)	50	(1) Website for computer-assisted self-management(CASM): goal setting, monitoring (HbA_1c_, BP, cholesterol), forum, education;^b,c^ (2) CASM+ social support (coach, group sessions)^b,c^	(3) Computer-based health risk appraisal, no key features of CASM	Behavior changes in diet, physical activity, medication adherence
My care team 2005 [45]	2-arm RCT; USA; 12 m	104 people with DM, HbA_1c_ ≥9.0% via veteran clinic	63.5 (7.0)	0.5	Website: self-management (glucose, BP), education, reminders (phone); care manager support	DM self-management training, usual care	HbA_1c_ and BP at 3, 6, 9, and 12 m
Mobile DM 2011 [32]	4-arm cluster RCT; USA; 12 m	26 physician practices with 163 people with DM and HbA_1c_ ≥7.5%	52.8 (8.1)	50	(2) Self-management via website + mobile phone, patient informs doctor;^b^ (3) 2 + doctor access to data; (4) 3 + advice from doctor^c^	(1) Care as usual	Change in HbA_1c_ over 1 year
Avdal 2011 [46]	2-arm RCT; Turkey; 6 m	122 people with DM2, Internet from clinic	51 (7.3)	51	Website: review risk profile, messaging to researcher, daily glucose monitoring	Education and usual care	HbA_1c_, attendance rates at outpatient clinic
Cho 2006 [47]	2-arm RCT; South Korea; 30 m	80 people with DM, Internet from clinic	53 (9)	39	Website: monitoring (glucose, medication, BP, weight, lifestyle), nurse feedback, medication alterations	Conventional note-keeping record system	HbA_1c_ and HbA_1c_ fluctuation index
Lorig 2010 [48]	3-arm RCT; USA; 6 m	761 people with DM2, Internet	54.3 (9.9)	73	Self-management website with peer support: lessons, action plans, bulletin board, messaging	Care as usual	HbA_1C_ level at 6 and 18 months
Grant 2008 [49]	2-arm cluster RCT; USA; 12 m	244 people with DM, HbA_1c_ >7.0% from 11 primary clinics	56.1 (11.6)	49	Online personal health record: education, diabetes care plan, agenda, messaging, prescription refills	Access to general website Patient Gateway	Changes in HbA_1c_, BP, and LDL cholesterol
McMahon 2012 [50]	3-arm RCT; USA; 12 m	151 people with DM, HbA_1c_>8.5% from veteran health services	60.2 (10.8)	5	(1) Self-monitoring via phone (BP, glucose); (2) website: self-monitoring (BP, glucose), education, support by care managers^b,c^	(3) Website with links to other DM websites; usual care	Change in HbA_1c_ and BP over time
Ralston 2009 [51]	2-arm RCT; USA; 12 m	83 people with DM2, HbA_1c_≥7.0% and Internet from clinic: 65% with 2 CV risk factors	57.3 (—)	52	Electronic medical record: self-monitoring (glucose, exercise, diet, medication), support by care manager, usual care visits	Usual care visits	Change in HbA_1c_
Kwon 2004 [52]	2-arm RCT; South Korea; 3 m	110 people with DM2, Internet from clinic: 27% hypertension	54.1 (9.1)	33	Website: self-monitoring (glucose), reminders, professor/nurse/dietician-support	Monthly visit to diabetes specialist	HbA_1c_
EMPOWER-D 2013 [39]	2-arm RCT; USA; 12 m	415 people with DM and HbA_1c_ ≥7.5% from clinic	53.7 (10.2)	40	Online health record: risk estimation, self-monitoring (glucose, diet, exercise, BP), nurse support, own doctor informed	Usual care	HbA_1c_ at 12 m
REDEEM 2013 [53]	3-arm RCT; USA; 12 m	392 people with DM2, Internet from community centers	56.1 (9.6)	54	(1) CASM website: goal setting; self-monitoring (HbA_1c_, BP, cholesterol); 8 phone calls;^b^ (2) Computer-assisted self-management + problem solving treatment (CASP): CASM + 8 sessions problem solving	Computer health risk appraisal, education, same phone calls as intervention	Diabetes distress; HbA_1c_, physical activity, medication compliance

^a^ Abbreviations: BP: blood pressure; CASM: computer-assisted self-management; CASP: computer-assisted self-management + problem solving treatment; CV: cardiovascular; DM: diabetes mellitus; DM2: type 2 diabetes mellitus; GP: general practitioner; HbA1c; glycated hemoglobin A1c; HDL: high-density lipoprotein; LDL: low-density lipoprotein.

^b^ For studies with more than 2 arms, this arm was used for all analyses.

^c^ For studies with more than 2 arms, this arm was used for the subgroup analysis on blended interventions.

**Table 2:** Characteristics of the studies included for the systematic review: interventions targeting blood pressure.^a^

Study	Setting and study length	Participants	Age (years), mean (SD)	Sex (% female)	Intervention	Control	Primary; secondary outcomes
e-BP 2008 [29]	3-arm RCT; USA; 12 m	778 people with Internet, hypertension, from GPs: 61.1% obese	59.1 (8.5)	52	(1) Website: BP self-monitoring;^b^ (2) 1 + pharmacist support^c^	General website: personal medical record	Change in diastolic, systolic and mean BP
Nolan 2012 [54]	2-arm RCT; Canada; 4 m	387 people with hypertension via website: 41% obese	56.5 (7.4)	59	BP action plan website: assessing motivational readiness, advice, feedback, education	E-newsletters	Change in diastolic and systolic BP, and pulse pressure
Bove 2013 [55]	2-arm RCT; USA; 6 m	241 people with elevated BP from 2 clinics	59.6 (13.6)	65	Website + telephone system: education, self-monitoring (BP, weight, exercise), online nurse support, doctor informed	Provision of data from initial assessment, usual care	Proportion of participants with controlled BP at 6 m
Madsen 2008 [56]	2-arm RCT; Denmark; 6 m	236 people with hypertension from 10 GPs	55.9 (11.7)	50	Website: self-monitoring (BP), feedback from own doctor by email	Usual care	Change in ambulatory systolic BP -at 6 m
Magid 2013 [57]	2-arm RCT; USA; 6 m	348 people with hypertension from 10 clinics	60 (11)	40	Written educational material, website: self-monitoring (BP), pharmacist support, doctor informed, reminders	Written education material, usual care	Proportion of participants with controlled BP at 6 m
McKinstry 2013 [38]	2-arm RCT; Scotland; 6 m	401 people with hypertension from 20 GPs	60.7 (11.2)	40	Telemonitoring unit + website: self-monitoring (BP), feedback from own doctor	Usual care	Mean ambulatory BP at 6 m
Thiboutot 2013 [58]	2-arm cluster RCT; USA; 12 m	500 patients with elevated BP from 54 GPs	60.5 (11.9)	58	Website: self-monitoring (BP, medication), feedback, reminders	Different prevention website (eg, breast screening)	BP control at 12 m

^a^ Abbreviations: BP: blood pressure; GP: general practitioner.

^b^ For studies with more than 2 arms, this arm was used for all analyses.

^c^ For studies with more than 2 arms, this arm was used for the subgroup analysis on blended interventions.

**Table 3:** Characteristics of the studies included for the systematic review: interventions targeting weight loss and weight loss maintenance.^a^

Study		Setting and study length	Participants	Age (years), mean (SD)	Sex (% female)	Intervention	Control	Primary; secondary outcomes
**Weight loss**								
	Appel 2011 [35]	3-arm RCT; USA; 24 m	415 people with obesity, ≥1 CV risk factor, Internet from 6 primary clinics	54 (10.2)	64	(1) Website + mobile coach support: education, self-monitoring (weight, diet, exercise), reminders, doctor informed;^b,c^ (2) 1 + in-person support	1 (or 2) meetings with coach; brochure with websites for weight loss	Change in weight from baseline to 24 m
	Bennett 2012 [28]	2-arm RCT; USA; 24 m	365 obese people with hypertension from 3 clinics	54.6 (10.9)	69	Website/interactive voice response system: self-monitoring weight, setting, coach support (phone), group sessions, education	Self-help booklet	Change in weight at 24 m
	Bennett 2010 [59]	2-arm RCT; USA; 3 m	101 obese people with hypertension, Internet from clinic	54.4 (8.1)	48	Website: goal setting, self-monitoring, behavioral skills education, forum, coach support (online, phone, face-to-face)	Folder on healthy weight, usual care	Change in weight at 12 weeks
	Kraschnewsky 2011 [60]	2-arm RCT; USA; 3 m	100 overweight people, Internet via flyers/Internet	50.3 (10.9)	70	Website: target body weight, monitoring, behavioral tips, videos, weight loss plan, tailored feedback, reminders	Wait list, people got access to website after 12 weeks	Weight loss
	Webber^d^ 2008 [61]	2-arm RCT; USA; 4 m	66 women, BMI 25-40, Internet from advertisements	50.0 (9.9)	100	Website: weight loss tips, lessons, message board, self-monitoring (weight, diet), chat sessions	All features of intervention except for online chat sessions	Not defined; weight, BMI, diet, exercise
	E-LITE 2013 [36]	3-arm RCT; USA; 15 m	241 people with a BMI ≥25, metabolic syndrome from 1 clinic	52.9 (10.6)	47	(1) Website + 12 lifestyle classes;^c^ (2) website: self-monitoring (weight, exercise), messaging, DVD with lifestyle classes^b^	Usual care	Change in BMI from baseline to 15 m
	POWER 2014 [62]	4-arm RCT; UK; 12 m	179 people with BMI ≥30 kg/m^2^ or ≥28 kg/m^2^ + CV risk factors from 5 GPs	51.2 (13.1)	66	(1) Website: 12 self-management sessions monitoring (weight), nurse support (email);^b,c^ (2) 1 + 3 nurse contacts; (3) 1 + 7 nurse contacts	Usual care	Weight at 12 m
**Weight loss maintenance**								
	Stop Regain 2008 [41]	3-arm RCT; USA; 18 m	314 people with 10% weight loss in 2 years, via advertisements	51 (10)	81	(1) Website: self-monitoring, email counseling, experts chat;^b^ (2) face-to-face: self-monitoring via phone, weekly group sessions	(3) Newsletters	Weight gain at 18 m
	WLM 2008 [40]	2-phase 3-arm RCT; USA; 30 m	1032 people with ≥4 kg previous weight loss, hypertension, Internet via university/ medicare	55.6 (8.7)	63	(1) Website: goal setting, action plans, self-monitoring (weight, PA, diet), education, bulletin board, reminders, support (email/phone);^b^ (2) personal contact (phone +face-to-face)	Printed lifestyle guidelines, 1 visit with coach	Change in weight

^a^ Abbreviations: BMI: body mass index; CV: cardiovascular; GP: general practitioner; PA: physical activity.

^b^ For studies with more than 2 arms, this arm was used for all analyses.

^c^ For studies with more than 2 arms, this arm was used for the subgroup analysis on blended interventions.

^d^ Control arm consists of same interactive Internet platform as intervention arm.

**Table 4:** Characteristics of the studies included for the systematic review: interventions targeting physical activity and cholesterol.^a^

Study		Setting and study length	Participants	Age (years), mean (SD)	Sex (% female)	Intervention	Control	Primary; secondary outcomes
**Physical activity**								
	Richardson^b^ 2010 [63]	2-arm RCT; USA; 4 m	324 patients from clinic: 12% CHD, 20% DM2, 62% BMI >30	52.0 (11.4)	65	Website as control + online community forum	Website: pedometer, tailored feedback	Change in average daily step count, patient attrition
	Reid 2011 [30]	2-arm RCT; Canada; 12 m	223 patients with a recent CHD event, Internet via 2 cardiac centers	56.4 (9.0)	16	Website: tutorials, exercise plans, self-monitoring, specialist support	Usual care, education booklet	Mean steps per day
	Ferney 2009 [64]	2-arm RCT; Australia; 6 m	106 inactive residents: 58% overweight	52.0 (4.6)	72	Website: behavioral strategies, goal setting, self-monitoring, advice, bulletin board, news	Website with minimal interactivity	Not defined; physical activity, website use
	Active after 55 2013 [65]	2-arm RCT; USA; 3 m	405 sedentary people with Internet via senior centers/websites	60.3 (4.9)	69	Website: education, goal setting, exercise planning, 11 online exercise lessons, self-monitoring, reminders	No access to the intervention	Not defined; physical activity, BMI
	HEART 2014 [37]	2-arm RCT; New Zealand; 6 m	171 people with stable CHD, Internet from 2 hospitals	60.2 (9.2)	19	Exercise prescription, behavioral strategies, Website: videos, self-monitoring (exercise), education, reminders	Usual care	Change in peak oxygen uptake from baseline to 6 m
	Philips Direct Life 2013 [66]	2-arm RCT; Netherlands; 3 m	235 inactive people with Internet through local media	64.8 (2.9)	41	Website: goal setting, self-monitoring (exercise), e-coach feedback	Waitlist control	Change in physical activity
	Suboc 2014 [67]	3-arm RCT; USA; 3 m	114 sedentary people through media and Internet	63.0 (7.0)	34	(1) Pedometer; (2) website + pedometer: exercise strategies, goal setting, self-monitoring (exercise) feedback, forum^c^	No intervention	Endothelial function; vascular stiffness, step count, exercise
	Peels 2013 [68]	5-arm cluster RCT; Netherlands; 12 m	2140 people from 6 municipal regions, ±50% overweight	63.2 (8.4)	51	(1) Printed feedback report; (2) 1 + local exercise tips; (3) Web-based feedback report; (4) 3 + local exercise tips^c^	Waitlist control	Physical activity
**Cholesterol**								
	Bloch^b^ 2006 [69]	3-arm RCT; USA; 6 m	171 employees with increased cholesterol, DM or CHD	54.8 (9.4)	—	(1) Website + financial reward; (2) website + 4 classes, nurse support (phone)	Website, 10-year CVD score, monitoring, goals, tailored info	LDL cholesterol change at 6 m
	Live well 2013 [70]	2-arm RCT; USA; 3 m	61 people with LDL cholesterol ≥3.37 mmol/L, Internet from primary clinics	52.0 (12.8)	75	Web-based rate-your-plate assessment, written educational material, Website: goal setting, self-monitoring, reminders	Web-based rate-your-plate assessment	Not defined; cholesterol, weight, Framingham risk score

^a^ Abbreviations: BMI: body mass index; CHD: coronary heart disease; CVD: cardiovascular disease; DM: diabetes mellitus; DM2: type 2 diabetes mellitus; LDL: low-density lipoprotein.

^b^ Control arm consists of same interactive Internet platform as intervention arm.

^c^ For studies with more than 2 arms, this arm was used for all analyses.

**Table 5:** Characteristics of the studies included for the systematic review: interventions targeting multiple risk factors.^a^

Study	Setting and study length	Participants	Age (years), mean (SD)	Sex (% female)	Intervention	Control	Primary; secondary outcomes
Lindsay 2008 [71]	2-arm RCT; UK; 6 m	108 heart patients living in deprived areas	62.9 (6.0)	33	eHealth portal: glossary, education, local community links, discussion forum	No access to the eHealth portal	Not defined; behavior change (exercise, smoking, diet)
Heartcare II 2010 [72]	2-arm cluster RCT; USA; 30 m	282 patients with chronic heart disease needing nursing care	64.0 (12.7)	39	Personal health record: education, monitoring, communication, goal setting, email, bulletin board	Usual care as the home care agencies use to provide	Satisfaction with nursing care
Hughes 2011 [73]	3-arm RCT; USA; 12 m	423 senior university employees with Internet, 32% overweight, 46% obese	51.0 (7.0)	82	(1) Coach for Web-based risk assessment, lifestyle plan, email, phone or in-person contact;^c^ (2) website: risk profile assessment, advice, goal setting, action planning^b^	Printed list of health promotion programs	Not defined; diet, exercise, weight
Southard 2003 [26]	2-arm RCT; USA; 6 m	104 patients with CHD or heart failure from 10 hospitals, 200 GPs, adverts	62.3 (10.6)	25	Website + nurse: education, self-monitoring, discussion group, links contact (email, phone or mail), dietician	Usual care	Not defined; weight, exercise, BP, lipid profile, new CV events
Winett 2007 [74]	3-arm cluster RCT; USA; 16 m	14 churches with 1071 members: 57% overweight, 60% sedentary	51.4 (15.7)	67	(1) Website: education, goal setting, pedometer;^b^ (2) 1 + pulpit support^c^	Waitlist condition	Nutrition improvement, physical activity
Vernooij 2012 [27]	2-arm RCT; Netherlands; 12 m	330 patients with CVD, 2 risk factors, Internet via 2 hospitals	59.9 (8.4)	25	Website: risk profile, self-monitoring (BP, cholesterol), treatment goal, nurse support, news, medication changes	Usual care by specialist or GP, receiving baseline risk profile	Relative change in Framingham heart risk score after 1 year
Verheijden 2004 [75]	2-arm RCT; Canada; 8 m	146 people with increased CV risk, Internet from 14 GPs	63.0 (10.5)	45	Website: tailored information, diet tool, bulletin board	Usual care	Not defined; BMI, BP, lipid profile
Ross 2004 [33]	2-arm RCT; USA; 12 m	107 patients with heart failure, Internet via clinic	56.0 (-)	23	Online medical record (clinical notes, laboratory reports, test results), education, nurse support	Usual care	Change in self-efficacy domain
Bove 2011 [76]	2-arm RCT; USA; 12 m	465 people with CVD risk >10% via community, clinics, churches	61.0 (10.0)	46	Online telemedicine system: laboratory and medication review, self-monitoring (BP, weight, pedometer), feedback, education, own doctor involved	4-months meetings with nurse: review data from logbooks	Reduction in Framingham 10-year CVD risk score
Keyserling 2014 [31]	2-arm RCT; USA; 12 m	385 people with CHD risk score ≥10% but no CVD from 5 GPs	62.0 (7.8)	48	Website: CHD risk calculator, advice, education, action planning, goal setting.	Same CHD risk calculator, but in-person and by phone	Framingham 10-year CHD risk score at 4 and 12 m
Zullig 2014 [77]	2-arm RCT; USA; 3 m	96 people with CVD or DM from primary clinics	36.1 (12.2)	67	CVD risk assessment, website: 6 modules with risk assessments, goal setting, education	Printed information on CVD	Not defined; Framingham 10-year CVD risk score, BMI, smoking status, systolic BP
Activate your Heart 2014 [78]	2-arm RCT; UK; 6 m	95 people with stable angina, Internet from 9 GPs	66.2 (9.2)	25	Website: CVD risk assessment, education, goal setting, self-monitoring, email/chat with experts	Usual care with GP	Change in step count at 6 weeks and 6 m
e-Care 2014 [79]	2-arm RCT USA 6m	101 people with BMI >26, elevated BP via electronic health records	56.9 (7.0)	42	Website + dietician: CVD risk assessment, goal setting, action planning, self-monitoring (weight, BP, physical activity, diet)	Usual care, printed report for patient and doctor	Change in systolic BP, weight and 10-year CVD risk score
Greene 2012 [80]	2-arm RCT; USA; 6 m	513 employees + families 45% overweight and 48% obese	60% older than 50 years	79	Printed lifestyle guide, website: online social network, self-monitoring (weight, exercise), goal setting, feedback	Printed lifestyle guide	Not defined; physical activity, weight, lipid profile
Holbrook 2009 [81]	2-arm cluster RCT; Canada; 12 m	46 GPs with 511 people with DM, ≥1 CV risk factor	60.7 (12.5)	49	Personal Web-based profile overview for DM/CVRM care, automated telephone reminders, summary for doctor, doctor involved	Usual care	Composite score for process of care
Diabetes in Check 2014 [82]	2-arm RCT; Australia; 9 m	436 people with DM, Internet via DM network	58.2 (10.3)	48	Website: self-monitoring (exercise) goal setting, education, discussion board	General website with home page and contacts page only	Not defined; physical activity, BMI

^a^ Abbreviations: BMI: body mass index; BP: blood pressure; CHD: coronary heart disease; CV: cardiovascular; CVD: cardiovascular disease; CVRM: cardiovascular risk management; DM: diabetes mellitus; GP: general practitioner.

^b^ For studies with more than 2 arms, this arm was used for all analyses.

^c^ For studies with more than 2 arms, this arm was used for the subgroup analysis on blended interventions.

#### Glycated Hemoglobin A1c

**Figure 4:**
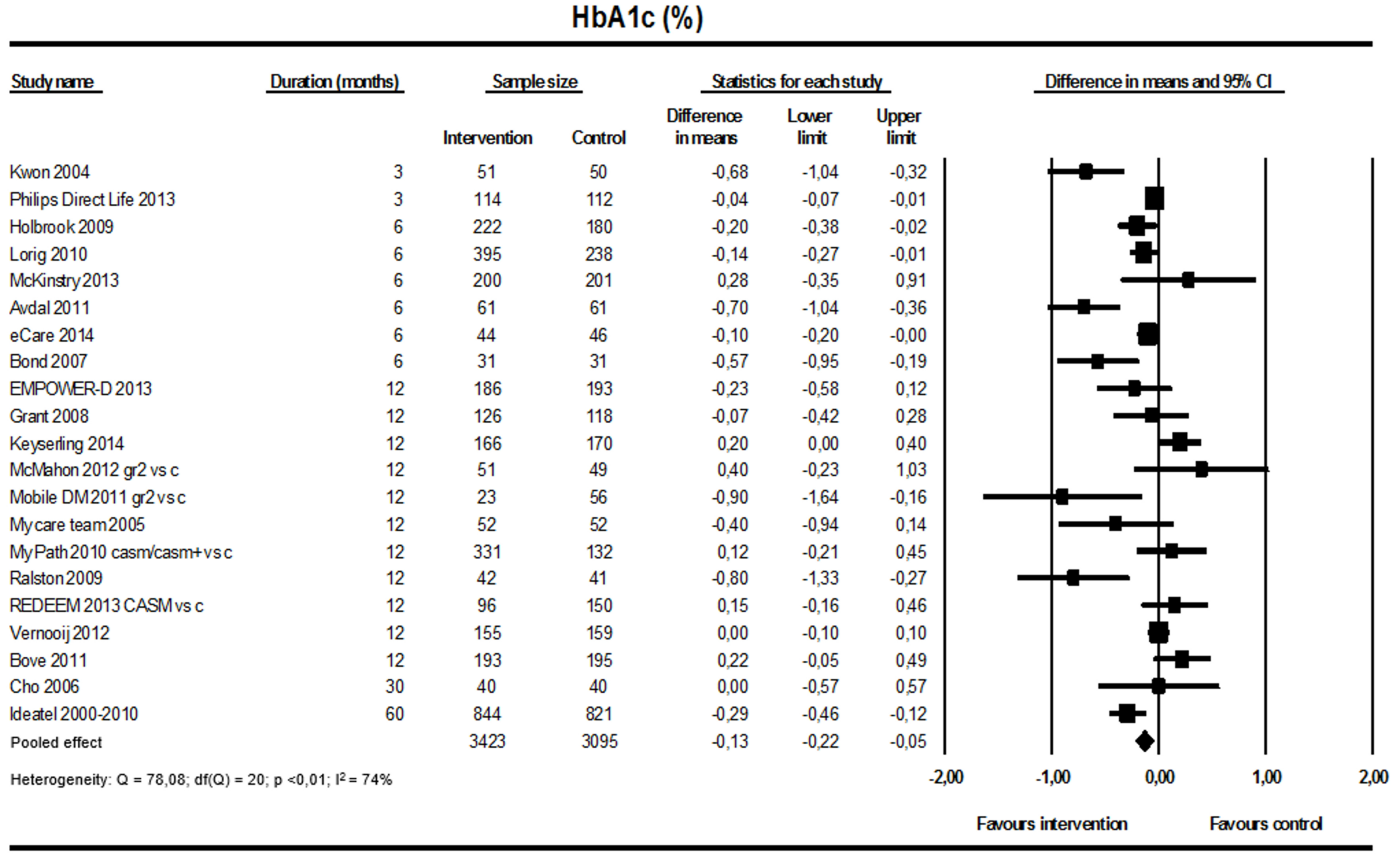
Effect on glycated hemoglobin (21 studies).

A significant reduction in HbA_1c_ level favoring the intervention among patients with type 2 diabetes mellitus was found (21 studies; n=6518; Figure 4). The weighted mean difference for HbA_1c_ was –0.13% (95% CI –0.22 to –0.05; I^2^=74%). The jackknife procedure did not reveal one particular study responsible for high heterogeneity.

#### Weight

**Figure 5:**
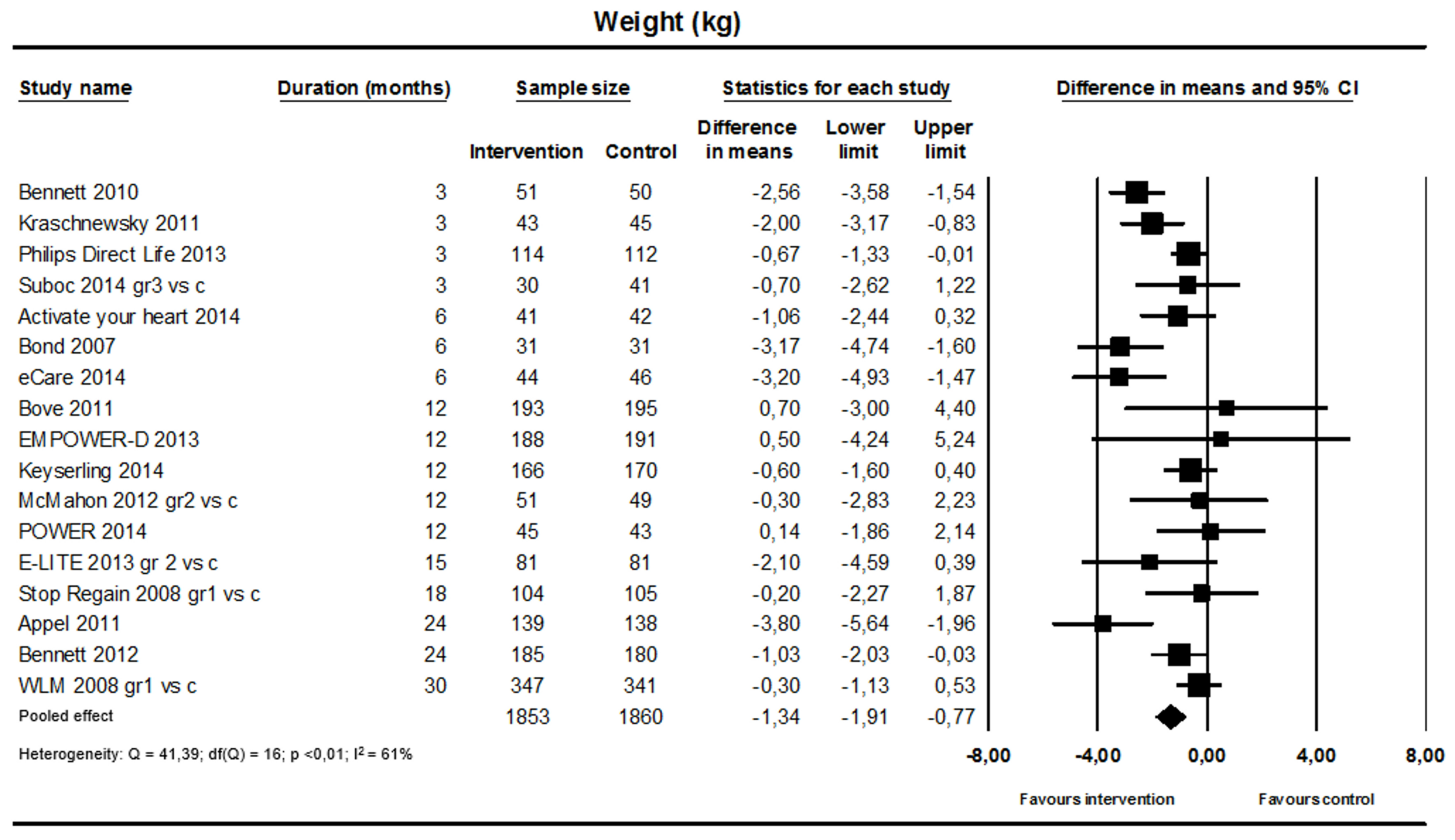
Effect on weight (17 studies).

Fifteen studies tested interventions for weight loss and two studies tested interventions for maintenance of weight loss. The pooled analysis (17 studies; n=3713; Figure 5) showed a significant reduction in weight favoring the intervention (weighted mean difference –1.34 kg, 95% CI –1.91 to –0.77; I^2^=61%). A sensitivity analysis leaving out the two studies on weight loss maintenance resulted in a similar effect size and level of heterogeneity. The jackknife procedure identified three studies contributing considerably to heterogeneity [35,42,59].

#### Low-Density Lipoprotein Cholesterol

**Figure 6:**
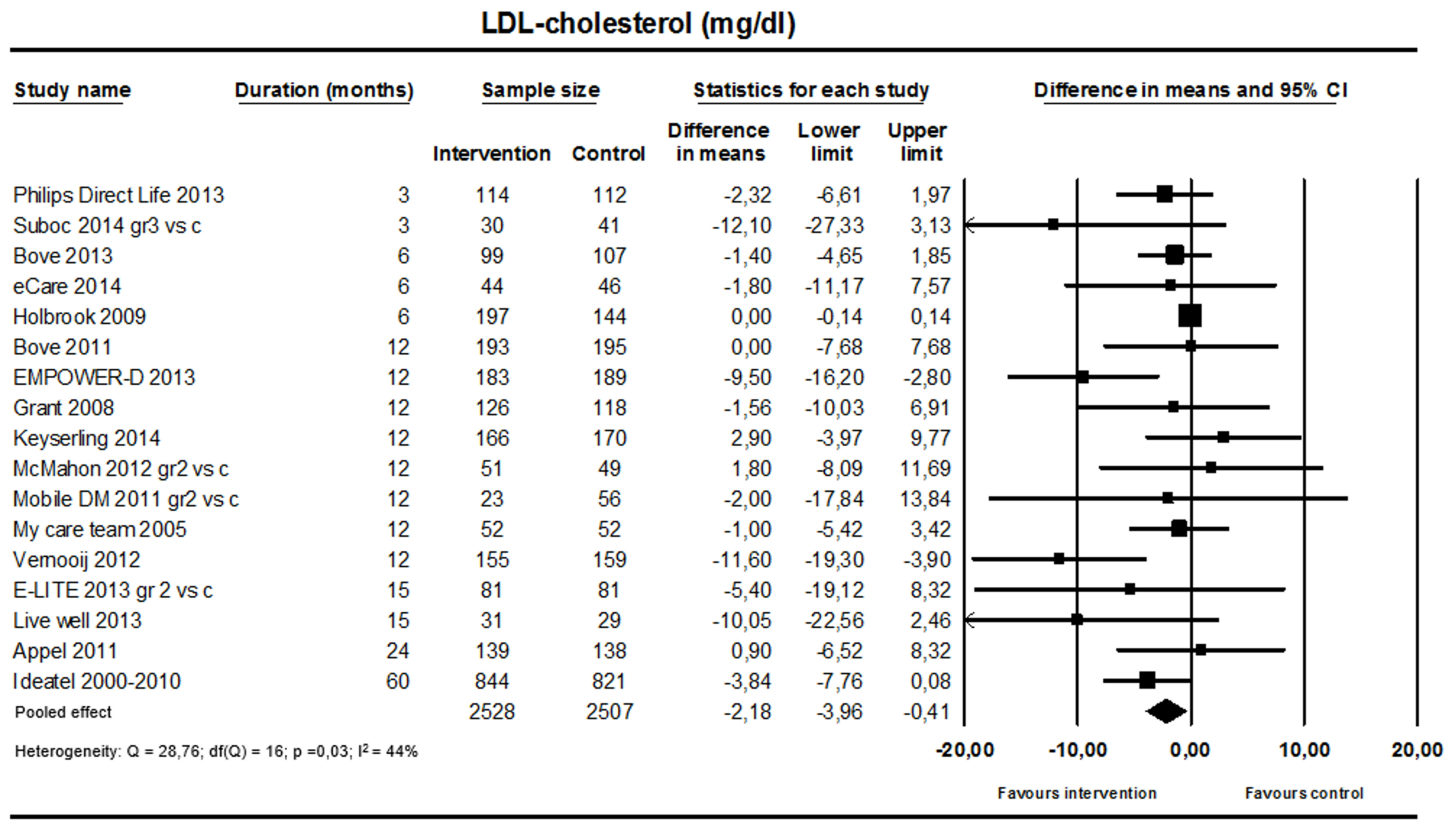
Effect on low-density lipoprotein cholesterol (17 studies).

A small but significant reduction in LDL cholesterol favoring the intervention was found (17 studies; n=5035; Figure 6; weighted mean difference –2.18 mg/dL, 95% CI –3.96 to –0.41; I^2^=44%).

#### Physical Activity

**Figure 7:**
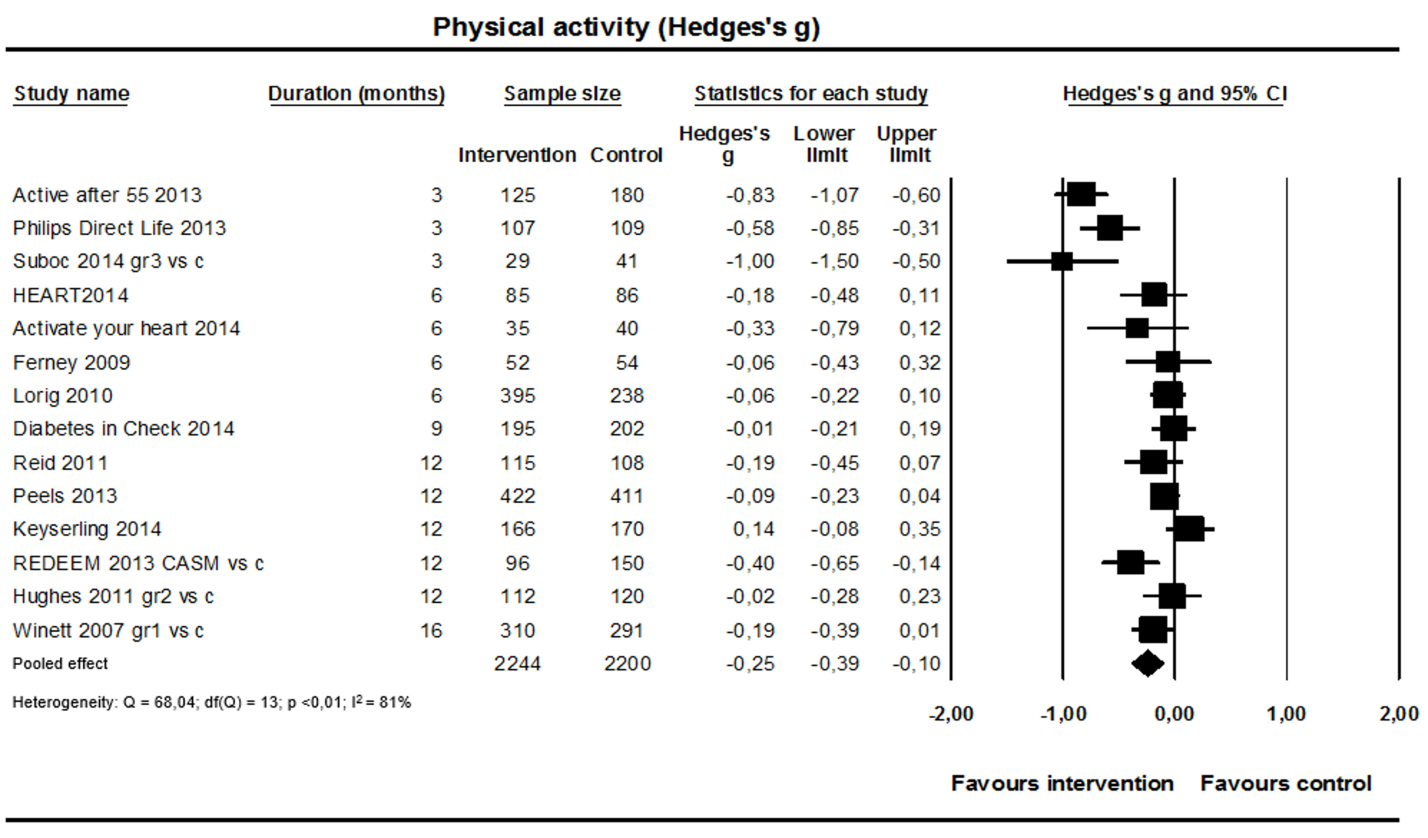
Effect on physical activity (14 studies).

Fourteen studies (n=4444; Figure 7) reported the effect on physical activity. Eight studies used self-reported physical activity levels in minutes per week, five studies used daily step counts obtained from pedometers, and one study measured physical activity with accelerometers. Because of the differences in measurement instruments, we calculated standardized mean differences. A small significant difference in increase of physical activity levels was found in favor of the intervention (weighted standardized mean difference 0.25, 95% CI 0.10-0.39; I^2^=81%), but heterogeneity was high. The jackknife procedure identified one study [65] driving a substantial part of heterogeneity; without this study, I^2^ was 68%.

### Effect of Web-Based Interventions on Overall Cardiovascular Risk Profile, Cardiovascular Morbidity, and Mortality

#### Cardiovascular Composite Scores

**Figure 8:**
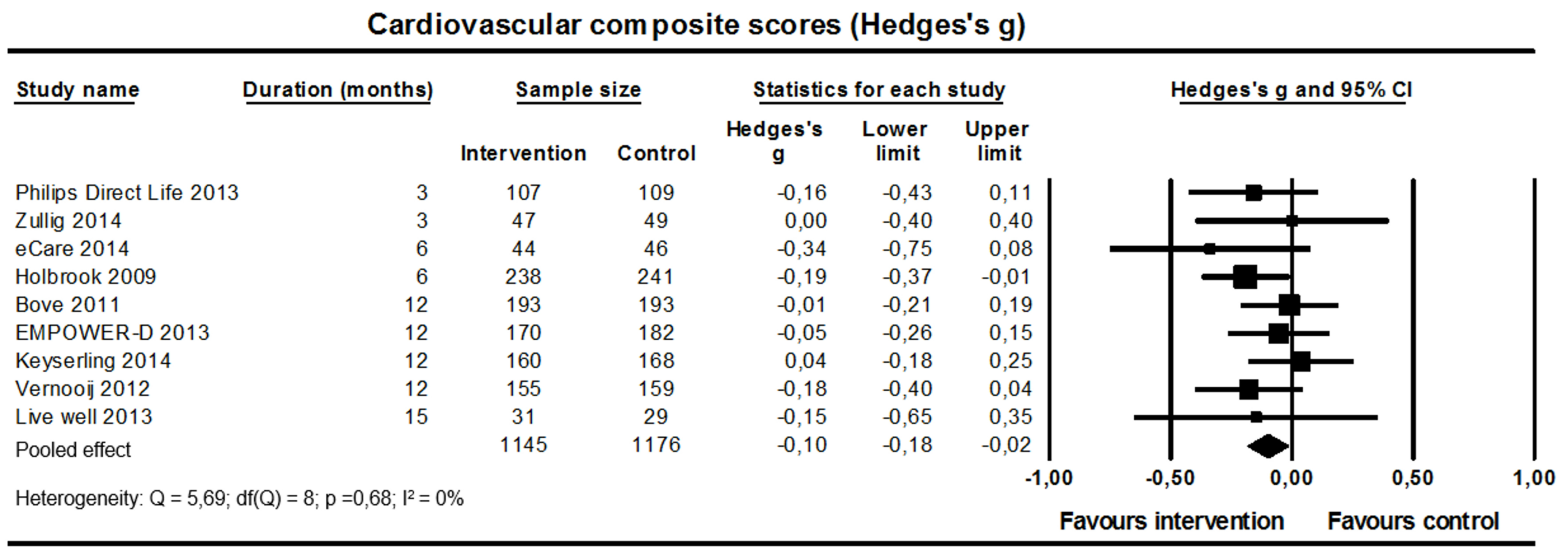
Effect on cardiovascular composite scores (9 studies).

Nine studies (n=2321; Figure 8) reported a cardiovascular composite score. Five studies reported the Framingham 10-year cardiovascular disease risk score, three studies reported the Framingham 10-year coronary heart disease risk score, and one study reported a clinical composite score based on number of cardiovascular risk factors on target (BP, HbA_1c_, body mass index, LDL cholesterol, physical activity, albuminuria, foot ulcers, and smoking). Because of the differences between the composite scores, we calculated standardized mean differences. A small significant improvement of the cardiovascular composite scores was found (weighted standardized mean difference –0.10, 95% CI –0.18 to –0.02; I^2^=0%).

#### General Effect of Web-Based Interventions on Cardiovascular Risk Factors

**Figure 9:**
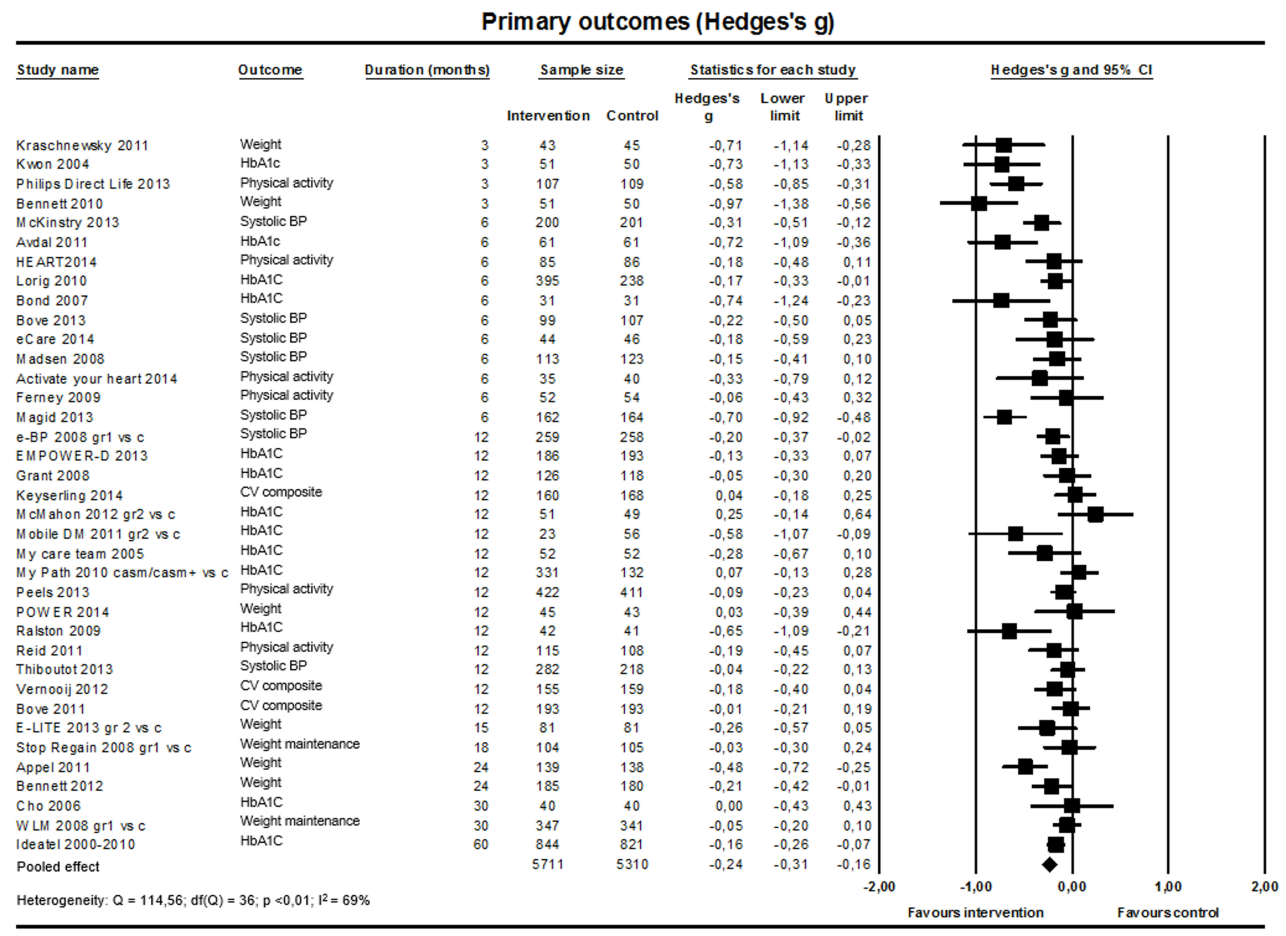
General effect on primary outcomes (37 studies).

Finally, we pooled the primary outcomes of the 37 studies (n=11,021; Figure 9) that defined a primary outcome (systolic BP: 7 studies; HbA_1c_: 13 studies; weight: 8 studies; physical activity: 6 studies; cardiovascular composite score: 3 studies). The weighted standardized mean difference was –0.24 (95% CI –0.31 to –0.16; I^2^=69%) in favor of the intervention. The jackknife procedure revealed that one study [57] somewhat influenced the heterogeneity; without this study, heterogeneity dropped to 64%. The funnel plot (Multimedia Appendix 5) indicated that small studies reporting large effects might be overrepresented. The Egger’s test confirmed that the funnel plot was not symmetrical (*P*=.01).

#### Cardiovascular Morbidity and Total Mortality

**Figure 10:**
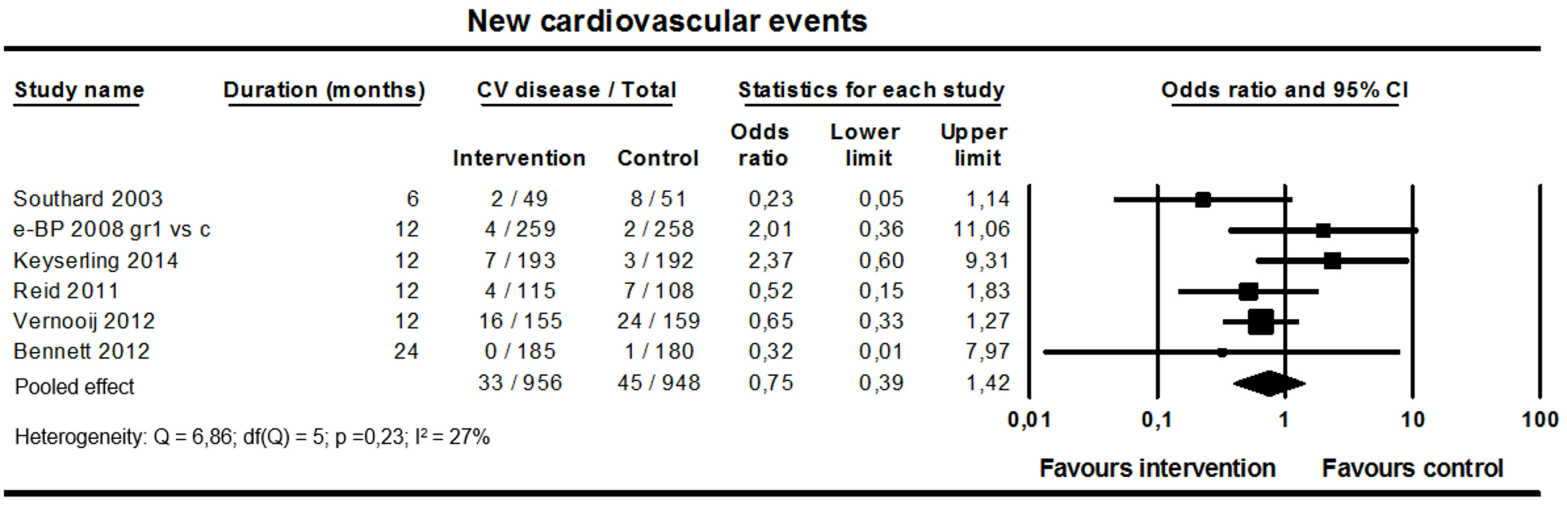
Effect on cardiovascular event rates (6 studies).

Six studies (n=1904; 1 short-term and 5 long-term studies) reported on cardiovascular event rates. The mean length of the studies was 13 months (range 6-24 months). The pooled analysis showed no difference in rate between groups (pooled OR 0.75, 95% CI 0.39-1.42; I^2^=27%; Figure 10). Total mortality rates were reported in 13 studies; in five studies, no deaths occurred and in the other eight studies, there were no differences between groups.

**Figure 11:**
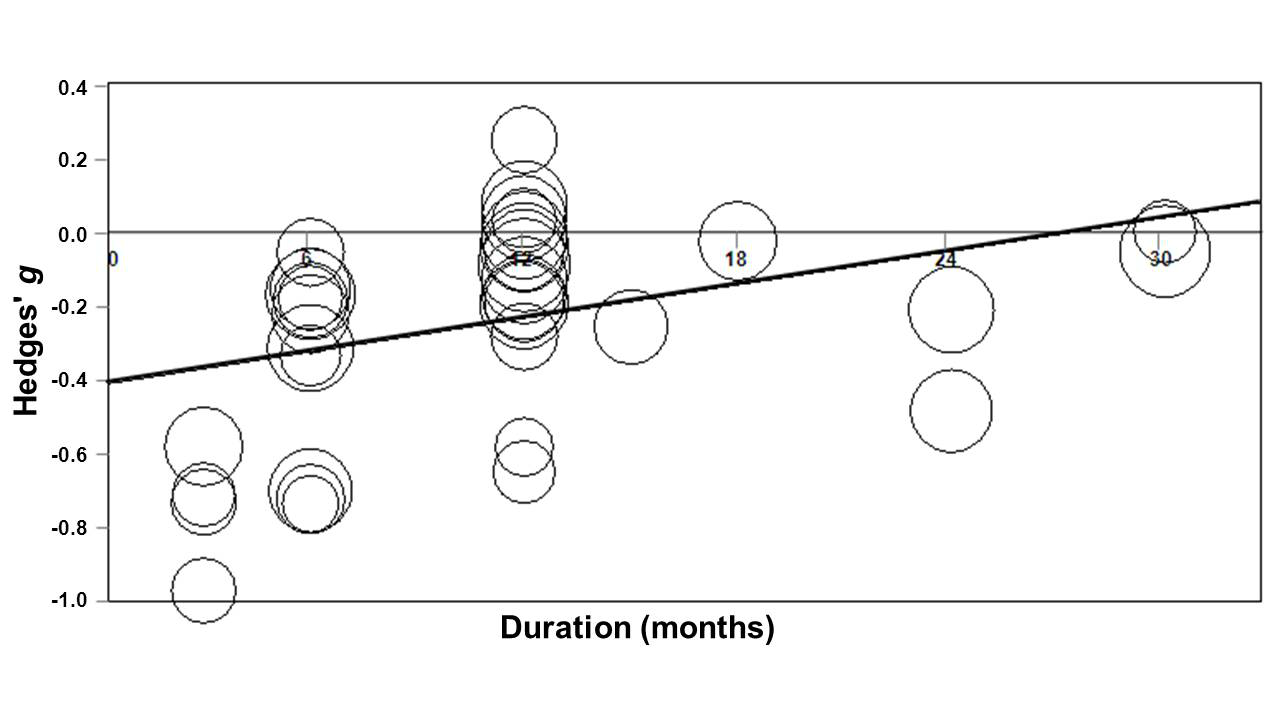
Association between study duration and effect size (Hedges' g). One outlier study (Ideatel) was removed from analysis.

### Subgroup Analyses

Results are summarized in Table 6. Within the analysis of pooled primary outcomes, the intervention effect was more pronounced in the short-term studies (15 studies; n=2934; standardized mean difference –0.43, 95% CI –0.57 to –0.29; I^2^=69%) than in the long-term studies (22 studies; n=8087; standardized mean difference –0.12, 95% CI –0.19 to –0.06; I^2^=41%). The same pattern was found for all other outcomes except for LDL cholesterol (Multimedia Appendix 6). There were no substantial differences in effect size between studies on primary prevention versus secondary prevention. To further explore the studies targeting primary prevention, we compared studies with populations of relatively low age (not all participants older than 50 years, n=29) with studies with populations of older age (all participants older than 50 years, n=4). The pooled effect size was larger for the studies with older participants (Hedges’ *g*=–0.30) than for the studies with relatively younger participants (Hedges’ *g*=–0.23), but the confidence intervals overlapped largely. We repeated the analysis of pooled primary outcomes on the sample of studies testing an Internet-only and a blended intervention. The intervention effect was more pronounced in the sample of blended studies (26 studies; n=7538; standardized mean difference –0.33, 95% CI –0.43 to –0.22; I^2^=78%) compared to the sample of Internet-only studies (14 studies; n=4280; standardized mean difference –0.15, 95% CI –0.23 to –0.07; I^2^=40%).

**Table 6:** Subgroup analyses within the analysis of standardized primary outcomes.

Subgroup		N of studies	Hedges’ *g*	95% CI	I^2^
**Duration** ^a^					
	Short (<12 months)	15	–0.43	–0.57, –0.29	69%
	Long (≥12 months)	22	–0.12	–0.19, –0.06	41%
**Type of prevention** ^a^					
	Primary (including diabetes control)	33	–0.25	–0.32, –0.17	72%
	Secondary	4	–0.20	–0.34, –0.06	0%
**Primary prevention: age subgroups** ^b^					
	Not all older than 50 years	29	–0.23	–0.33, –0.14	72%
	All older than 50 years	4	–0.30	–0.51, –0.09	80%
Internet only vs control^c^		14	–0.15	–0.23, –0.07	40%
Blended vs control^d^		26	–0.33	–0.43, –0.22	79%

^a^ Subgroup analysis performed in the sample of studies that was used for the analysis of primary outcomes.

^b^ Subgroup analysis performed on the sample of studies that targeted primary prevention (including diabetes control).

^c^ Subgroup analysis performed on the sample of studies that evaluated an Internet-only intervention. In case a study tested multiple arms, the appropriate arm was included in the analysis.

^d^ Subgroup analysis performed on the sample of studies that evaluated a blended intervention. In case a study tested multiple arms, the appropriate arm was included in the analysis.

### Meta-Regression

Because of the fairly consistent finding that treatment effects were higher in short-term studies than in long-term studies, we performed a mixed effects meta-regression to explore the association between study duration and effect size. The effect size seemed to become smaller in studies with longer follow-up, although the association was not significant (Hedges’ *g*=–0.321+0.006*months; *P*=.07). After removal of one outlier study [34] that had a very long follow-up (5 years), the effect size significantly decreased over time in studies lasting 3 to 32 months (Hedges’ *g*=–0.415+ 0.015*months; *P*=.008; Figure. 11).

### Sensitivity Analyses for the Risk-of-Bias Assessment

We performed sensitivity analyses for each of the six domains of bias assessed with the adapted Cochrane Risk of Bias Tool by comparing the standardized primary outcomes of the studies with the low risk and unknown/high risk of bias (Multimedia Appendix 7). There were no significant differences in pooled effect sizes in any of the domains except for the domain random sequence generation, in which the pooled effect was significantly larger in the subgroup of studies with unknown/high risk of bias.

## Discussion

In this systematic review and meta-analysis, we found for people with elevated cardiovascular risk, Web-based interventions lead to improvement of systolic and diastolic BP, HbA_1c_, weight, LDL cholesterol, physical activity levels, and cardiovascular risk composite scores. Only seven studies included participants all aged 50 years or older. Therefore, our conclusions apply for the population in middle age and beyond. Effects were more pronounced over the short term (study duration <12 months) and in studies that tested a blended intervention (combination of an Internet application and human support). We found no evidence for an effect on incident cardiovascular disease.

Our findings on single cardiovascular risk factors are consistent with conclusions of other meta-analyses in younger adult populations [19-21]. We found a significant reduction in systolic BP of 2.66 mmHg. A reduction of 3 mmHg in systolic BP can lead to an 8% reduction in annual stroke mortality rate and a 5% reduction in annual coronary heart disease mortality rate [83]. We found a reduction of LDL cholesterol of 2.18 mg/dL (converted=0.06 mmol/L). A reduction of 0.5 mmol/L in LDL cholesterol for at least 2 years can lead to a reduction in coronary heart disease events of 20% [6]. Theoretically, assuming a linear relation, a reduction of 0.06 mmol/L could lead to a 2.4% reduction of coronary heart disease events. Thus, the effects on Internet interventions on BP reduction and, to a lesser extent, LDL cholesterol reduction, can be clinically relevant at the population level if reductions are maintained. In addition, we evaluated the effect on the complete cardiovascular risk profile and prevention of cardiovascular disease, which has not been performed before. One other systematic review without meta-analysis that evaluated Internet interventions for lifestyle change in older people reported that interventions with multiple components are more effective than interventions with a single component [84].

We found that the beneficial effects of Web-based interventions decline over time and effects are larger when interventions are combined with human support. Decreasing adherence over time was reported in several studies included in our meta-analysis and could be an important contributor to the decreasing effect over time. We were unable to formally test this because information on adherence and engagement was only reported by 22 studies and definitions varied widely. The identified effect moderators are not specific to Web-based interventions for cardiovascular risk factors [85,86]. Maintenance of behavioral change is notoriously complex and best achieved in longer studies with intensive interventions, more face-to-face, and more follow-up contacts. However, such interventions lead to high attrition rates, probably reflecting selection of the most motivated participants [87]. A careful balance should be sought between effectiveness and implementability when designing cardiovascular risk management interventions, whether or not an Internet-based approach is used.

Our results do not show a beneficial effect of Web-based interventions on incident cardiovascular disease. Although the declining effect over time could play a role, more likely explanations for these findings are the limited follow-up time of the studies to detect these outcomes (mean length of the studies was 13 months) and the fact that these outcomes were not the primary focus of these studies. Because of the latter, data collection may not have been systematic and adjudication of the data by an independent committee may be lacking. Therefore, we cannot draw strong conclusions from these findings.

The results of this study should be interpreted with caution because of several limitations. The methodological quality of the studies was fair, but none of the studies was double blind, rendering them prone to performance bias. Only 20 studies had a blinded outcome assessment, so detection bias may also be present. Because the sensitivity analyses for the risk-of-bias assessment did not reveal significant differences between the low risk and unknown/high risk-of-bias subgroups, except for the domain of random sequence generation, we think that our findings have not been largely affected by these potential sources of bias. Another limitation is the substantial heterogeneity in several of the meta-analyses that is, in part, explained by two effect modifiers: study duration and intervention type. Patient groups with a higher burden have a larger window of opportunity for improvement potentially resulting in larger intervention effects [88], which could also have contributed to heterogeneity. We could not draw firm conclusions on the difference between primary and secondary prevention, because only four studies on secondary prevention were included in this analysis. Last, there is a potential for publication bias and small study bias. Most of the studies with small sample sizes reported large effects and similar studies with null findings did not appear in the funnel plots (Multimedia Appendix 5).

Strengths of our study are the comprehensive search strategy, the quantitative meta-analysis, and the assessment of the effect of Web-based interventions for all cardiovascular risk factors using both intermediate and clinical outcomes. Our search strategy was comprehensive because we used a broad definition of Web-based interventions and only excluded telemedicine and mobile phone interventions. It was not always possible to set Web-based interventions apart from telemedicine and mobile phone interventions. As long as the Web-based program was the main component of the intervention, we judged the study eligible for our systematic review. By pooling the effect sizes on all different cardiovascular risk factors, we aimed to assess the overall effect of an Internet-based approach for people with increased risk of cardiovascular disease. This approach provides insight into the overall potential of Internet-based interventions in this field. Although basic computer literacy as an inclusion criterion probably led to selection of participants with a relatively high socioeconomic status, several studies included in the meta-analysis focused on people from medically underserved areas. Therefore, the external validity of the results might be acceptable and may be generalizable to middle-aged to older primary care populations with an increased risk of cardiovascular disease.

Our results show that Web-based interventions can be effective in improving the cardiovascular risk factor profile of middle-aged and older people, but effects are modest and can only have clinical relevance on the population level if sustained over time. Considering the current interest and focus on eHealth by policy makers, funding agencies, and a myriad of research and patient organizations [89,90], it is important to evaluate the actual evidence base objectively. Unrealistic expectations of the effectiveness of Web-based interventions obscure the true challenges that have to be overcome first, including testing interventions that were designed specifically for older people, improving methodological robustness of studies, and improving sustainability of effects. On the macro level, trials can assess sustainability by prolonging follow-up, recording clinical events, and measuring surrogate cardiovascular outcomes (eg, BP, cholesterol levels, and weight) at multiple time points (eg, at 6, 12, 24, and 36 months). On the micro level, adherence should be evaluated by studying intervention usage through time with standardized evaluation methods. Sustainability is of particular importance because long-term effects are required for primary and secondary prevention to truly contribute to the prevention of cardiovascular disease. Web-based interventions combined with human support are more promising than Internet-only interventions.

## Appendix

Multimedia Appendix 1 Research protocol.

Multimedia Appendix 2 Comprehensive search strategy.

Multimedia Appendix 3 Data-extraction form.

Multimedia Appendix 4 Summary of the risk of bias assessment.

Multimedia Appendix 5 Funnel plots.

Multimedia Appendix 6 Subgroup analysis: study-duration.

Multimedia Appendix 7 Risk of bias assessment: sensitivity analyses for the domains of risk of bias.

## Abbreviations

BP: blood pressure
HbA1c: glycated hemoglobin A1c
IQR: interquartile range
LDL: low-density lipoprotein
PA: physical activity
RCT: randomized controlled trial
