# Supplementary material for: Web-Based Interventions Targeting Cardiovascular Risk Factors in Middle-Aged and Older People: A Systematic Review and Meta-Analysis
Source: J Med Internet Res. 2016 Mar 11;18(3):e55. doi: 10.2196/jmir.5218 (PMC4808240; doi:10.2196/jmir.5218)
Supplement: Multimedia Appendix 1 [file jmir_v18i3e55_app1.pdf]

Multimedia appendix 1: Research protocol

Systematic review 'Internet-interventions targeting cardiovascular risk factors and diseases' (working title)

CRL Beishuizen, E Richard, E P Moll van Charante and BCM Stephan 18-02-2013

1. Background

- Globally cardiovascular disease is the nr 1 cause of death and disability and in Europe is the nr 1 cause of death among >65 year olds (WHO and Eurostat)
- Ongoing rise of the older aged population in developed countries is resulting in larger demands and increased pressure on healthcare resources.
- Effective cardiovascular risk management contributes to prevention of cardiovascular disease and dementia (WHO Fact sheet N°317, September 2012 and [1]).
- Different kinds of treatment exists (pharmacological, non-pharmacological, single- and multi-component interventions). Drugs (e.g., anti-hypertensive medications, statins) are effective when studied in large RCT's [2, 3]. Multi-component intensive cardiovascular risk management programmes are widely implemented in primary care to address lifestyle (e.g., diet and physical activity). These intensive programs address multiple risk factors and are generally driven by a caregiver such as a nurse or a physician. Some of these programs have been proven to be effective in high-risk groups in research-settings [4, 5]. In real life, however, lifestyle improvement is not easy to reach, due to difficulties with motivation and adherence (reference).
- Need for new approaches to promote adherence/compliance to cardiovascular risk management programs.
- In the last decennium the internet as a vehicle for treatment has become a field of interest for researchers and policymakers.
- Elderly and the internet: in 2012, 42% of the European people aged 55-74 used the internet and this number is on the rise (Eurostat).
- Internet-interventions in various diseases, state of the art. In Psychiatry for example, many validated applications for depressive and anxiety disorders are already being used.
- Quite a few reviews about internet-interventions targeting one cardiovascular risk factor exist. Only few reviews about internet-interventions exist that simultaneously target multiple risk factors or multi-morbidity. Results of these reviews are mixed.
- In the last 2 years, large randomized controlled trials analyzing the effect of internet-interventions addressing multiple cardiovascular risk factors have been executed.
- The aim of this review is to evaluate the current evidence for efficacy of internet-interventions in elderly populations targeting cardiovascular risk factors and/or disease. We will compare internet-only with blended (e.g., internet combined with another intervention type such as caregiver support) care approaches.
  Cost-effectiveness will also be studied.

2. Review question and PICOS

Main question:

- In the elderly, are internet-interventions that target cardiovascular risk factors and/or disease effective on indirect (e.g., surrogate/intermediate) and clinical outcomes of cardiovascular disease?

Secondary questions:

- Are there differences in effectiveness between different types of interventions (e.g., purely internet vs. blended (such as combined with a caregiver for example, a nurse or physician)?
- What are the internet-intervention-usage statistics (as in: how often do people log on) and adherence rates?
- What is the effect of internet-interventions on outcomes such as quality of life and patient satisfaction?

PICOS

**P**	people with cardiovascular risk factors or with cardiovascular disease, >50 years
**I**	internet-intervention, web-based intervention
**C**	usual care
**O**	• Surrogate endpoints (blood pressure control, glucose control (HbA1c), BMI, cholesterol, physical activity and smoking)<br>• Clinical outcomes (new cardiovascular events or cardiovascular disease, disability related to cardiovascular disease and mortality)<br>• Adherence to drug therapy that is part of cardiovascular risk management<br>• Quality of life and patient satisfaction<br>• Internet-intervention-usage statistics and adherence rates
**S**	RCT, review (with or without meta-analysis)

3. Definitions

Cardiovascular risk factors were defined as: hypertension, diabetes mellitus, hypercholesterolemia, smoking, obesity, adiposity or overweight/obese (BMI>25 kg/m2), lack of physical exercise, a positive family history for cardiovascular disease and atherosclerosis or arteriosclerosis.

Cardiovascular disease was defined as: myocardial infarction, angina pectoris, heart failure, stroke or transient ischemic attack and peripheral arterial disease.

Internet-intervention was defined as: a web-based intervention, addressing the cardiovascular risk profile of the patient, providing one or more of the following: interactive educational material about cardiovascular risk factors and diseases, possibilities for self-monitoring and self-management of disease, prescription of medication, interactive feedback system with caregiver (physician, nurse or pharmacist), possibilities for goal setting with regard to risk profile, online community with peers.

4. Inclusion criteria

All randomized controlled trials evaluating internet-based interventions in participants aged 50 years and older targeting cardiovascular risk factors and/or disease. Sample size has to be above n=50. Included language was English. Patient characteristics, randomization, intervention, attrition and dropout have to be mentioned. If two publications describe one identical trial, data of the most relevant publication with the lowest chance of bias will be used.

In addition, we will study reviews and meta-analyses on this topic, in order to describe the existing body of evidence and to compare references listed in the articles with our search results and check for missed relevant articles.

Checklist selection criteria

1st selection on title/abstract:

- Internet-intervention for cardiovascular risk factors and/or disease
- Randomized controlled trial, review +/- meta-analysis
- N ≥ 50
- Population age ≥ 50 years

2nd selection on full text:

- Adequate randomisation
- Adequate description of patient characteristics (age and sex)
- Adequate description of intervention provided
- Duration of intervention ≥ 4 weeks and follow-up ≥ 3 months
- Adequate description of attrition and dropout rates

- At least one of the outcomes described above is studied

Exclusion criteria

- Design articles not reporting results
- RCT's about telemonitoring or telemedicine, defined as: using remote telecommunications to monitor the patients vital signs or disease symptoms or for diagnostics
- RCT's evaluating a computer-intervention without an internet-component

5. Search strategy

A pilot search in Pubmed and Google will be performed to encounter as many definitions of internet-intervention as possible.

The following databases will be consulted:

- Embase, Medline (including Pubmed), Cinahl
- Clinical trials.gov and WHO ICTRP

Searchterms (see also comprehensive list):

- Cardiovascular diseases (Mesh and ti/ab) and separate diseases specified (Mesh and ti/ab)
- Cardiovascular risk factors (Mesh and ti/ab) and separate risk factors specified (Mesh and ti/ab)
- Nutrition/diet/blood pressure control/glucose control/weight loss/exercise programs and related terms
- Internet-interventions and all possible names found in pilot search
- RCT, review, meta-analysis
- Age >50 as a filter will be considered

6. Study selection

Studies will be selected by screening of titles and abstracts using the 1st selection criteria defined above. Thereafter full papers will be assessed in detail using the 2nd selection criteria mentioned above.

Study selection will be done by two independent researchers. In the event of missing data the decision may be taken to contact authors of primary studies. Disagreements will be resolved by discussion or by a third independent investigator.

If necessary, the selection process will be piloted to further refine and clarify the inclusion criteria. This will be documented, just as the whole study selection process, reasons for exclusion and disagreements and solutions.

7. Quality assessment

Quality of the studies will be assed for the risk of bias and other general issues related to study quality using the following criteria, based on *Cochrane Handbook for Systematic reviews of Interventions* and *the Systematic Reviews Guidelines of the Centre of Reviews and Dissemination*:

- Study design appropriate for research objective
- Generation of allocation sequence
- Allocation concealment
- Double - blinding (not possible inherent to intervention), but were outcome assessors blind to treatment allocation?
- Description of intervention clear
- Statistical analyses, if a cluster-RCT was performed, was the statistical analysis appropriate?
- Completeness of data/ completeness of reporting
- Handling of dropouts/attrition
- Intention to treat analysis

8. Data-extraction

Data will be extracted using a data extraction form (provided). If necessary, during extraction, due to advancing insight, more details on one topic will be extracted from all included studies.

9. Data synthesis

We plan to use a quantitative approach to data-synthesis. If not enough quantitative data is provided, we will contact the authors of the primary study for more information.

Due to differences between studied interventions, we expect high heterogeneity between studies. Q and l-squared tests will be used to test for heterogeneity. If necessary, we will only combine primary studies using similar interventions or similar patient-groups for meta-analysis to reduce heterogeneity. If sufficient studies are identified sensitivity analysis will be carried out to take into account the influence of other variables e.g., study quality. Funnel plots will be used to assess for evidence of bias. Study quality will be evaluated using the criteria specified in the Cochrane Risk of Bias Tool assessing the following domains: measurement protocols, incomplete data outcome and selective reporting etc.

First, for surrogate outcomes (BP, BMI, HbA1c etc), we will calculate mean differences and their 95% confidence intervals. For the clinical endpoints, dichotomous in nature, we will calculate relative risks. For qualitative data (such as quality of life, adherence and patient satisfaction), a descriptive analysis will be given. Second, we will calculate pooled effect sizes using a fixed- and random effects model. Then, we will compare the models to test their robustness.

We can analyse what is the pooled effect size of a type of intervention on outcome (risk factor modelling or clinical outcome, or quality of life). We can also analyse what are the different effect sizes for a single risk factor or clinical outcome depending on type of intervention (pure internet or blended).

REFERENCES

1. Ritchie K, Carriere I, Ritchie CW, Berr C, Artero S, Ancelin ML. Designing prevention programmes to reduce incidence of dementia: prospective cohort study of modifiable risk factors. BMJ 2010;341:c3885.

2. Law MR, Morris JK, Wald NJ. Use of blood pressure lowering drugs in the prevention of cardiovascular disease: meta-analysis of 147 randomised trials in the context of expectations from prospective epidemiological studies. BMJ 2009;338:b1665.

3. Law MR, Wald NJ, Rudnicka AR. Quantifying effect of statins on low density lipoprotein cholesterol, ischaemic heart disease, and stroke: systematic review and meta-analysis. BMJ 2003;326:1423.

4. Angermayr L, Melchart D, Linde K. Multifactorial lifestyle interventions in the primary and secondary prevention of cardiovascular disease and type 2 diabetes mellitus--a systematic review of randomized controlled trials. Ann Behav Med 2010;40:49-64.

5. Ebrahim S, Taylor F, Ward K, Beswick A, Burke M, Davey SG. Multiple risk factor interventions for primary prevention of coronary heart disease. Cochrane Database Syst Rev 2011;CD001561.
