# Supplementary material for: Web-Based Interventions Targeting Cardiovascular Risk Factors in Middle-Aged and Older People: A Systematic Review and Meta-Analysis
Source: J Med Internet Res. 2016 Mar 11;18(3):e55. doi: 10.2196/jmir.5218 (PMC4808240; doi:10.2196/jmir.5218)
Supplement: Multimedia Appendix 2 [file jmir_v18i3e55_app2.pdf]

Multimedia appendix 2: Comprehensive search strategy

OvidSP Embase 1980 to Present

d.d.: 26-02-2013

Line#	Term
1	exp cardiovascular disease/
2	exp heart infarction/
3	exp cerebrovascular accident/
4	exp diabetes mellitus/
5	exp heart failure/
6	exp hypertension/
7	"smoking and smoking related phenomena"/ or exp cigarette smoke condensate/ or exp smoking/ or exp smoking regulation/ or exp tobacco smoke/
8	exp obesity/
9	exp sedentary lifestyle/
10	laziness/
11	exp hypercholesterolemia/
12	exp arteriosclerosis/
13	exp angina pectoris/
14	exp familial hypertrophic cardiomyopathy/
15	exp cardiovascular risk/
16	exp exercise/ or exp physical activity/
17	(physical adj activity).ti,ab,kw.
18	exp nutrition education/ or exp nutrition/
19	nutrition.ti,ab,kw.
20	exp diet/
21	exp blood pressure regulation/
22	(blood pressure adj (monitoring or regulation or control)).ti,ab,kw.
23	exp blood glucose monitoring/
24	exp home monitoring/
25	exp weight reduction/
26	(weight adj loss).ti,ab,kw.
27	((exercise or walking) adj program).ti,ab,kw.
28	(vascular adj2 risk).ti,ab,kw.
29	cholesterol.mp. or exp cholesterol/
30	(heart adj2 (infarct* or attack or faillure or disease)).ti,ab,kw.
31	(cardiovascular adj2 (risk or risks or diseas$)).ti,ab,kw.
32	stroke.ti,ab,kw.
33	(high adj blood pressure).ti,ab,kw.
34	hypertension.ti,ab,kw.
35	smoking.ti,ab,kw.
36	cerebrovascular.ti,ab,kw.
37	diabetes.ti,ab,kw.
38	sedentary.ti,ab,kw.
39	arteriosclerosis.ti,ab,kw.
40	cardiomyopathy.ti,ab,kw.
41	exp peripheral occlusive artery disease/
42	(peripheral adj2 arter* adj disease).ti,ab,kw.
43	exp atherosclerosis/
44	atherosclerosis.ti,ab,kw.
45	obese.tw.
46	overweight.tw.
47	or/1-46
48	exp telemedicine/ or exp Internet/
49	internet.ti,ab,kw.
50	(ehealth or e-health).ti,ab,kw.
51	telemedicine.ti,ab,kw.
52	telehealth.ti,ab,kw.
53	(mobile adj health).ti,ab,kw.
54	((internet or web or computer) adj2 intervention).ti,ab,kw.
55	(web-based adj2 tool).ti,ab,kw.
56	mhealth.ti,ab,kw.
57	(social adj media).ti,ab,kw.
58	exp social media/
59	facebook.ti,ab,kw.
60	apps.ti,ab,kw.
61	(ONLINE adj2 COMMUNITY).ti,ab,kw.
62	TELEMONITORING.ti,ab,kw.
63	web 2.ti,ab,kw.
64	mhealth.ti,ab,kw.
65	e-mail.ti,ab,kw. or exp e-mail/
66	((computer or internet) adj assisted adj therapy).ti,ab,kw.
67	web-site.ti,ab.
68	or/48-67
69	47 and 68
70	animal tissue/
71	animal model/
72	animal experiment/
73	exp invertebrate/
74	exp animals/
75	animal cell/
76	nonhuman/
77	or/70-76
78	human/ or normal human/ or human cell/
79	77 and 78
80	77 not 79
81	69 not 80
82	meta-analysis.tw.
83	systematic review.tw.
84	MEDLINE.tw.
85	pubmed.tw.
86	or/82-85
87	81 and 86
88	(random$ or crossover$ or placebo$ or (doubl$ adj blind$) or (singl$ adj blind$) or allocat$).ti,ab.
89	crossover-procedure/ or double-blind procedure/ or randomized controlled trial/ or single-blind procedure/
90	(Trial or comparison).ti.
91	or/88-90
92	81 and 91
93	limit 92 to (conference abstract or conference proceeding or "review")
94	92 not 93
95	limit 94 to yr="1995 -Current"
96	limit 87 to yr="1995 –Current"
