# Supplementary material for: Web-Based Interventions Targeting Cardiovascular Risk Factors in Middle-Aged and Older People: A Systematic Review and Meta-Analysis
Source: J Med Internet Res. 2016 Mar 11;18(3):e55. doi: 10.2196/jmir.5218 (PMC4808240; doi:10.2196/jmir.5218)
Supplement: Multimedia Appendix 3 [file jmir_v18i3e55_app3.pdf]

Multimedia appendix 3: Data-extraction form

Data extraction form Review 'Internet-interventions for cardiovascular risk management'

CRL Beishuizen, 26-04-2013

Source

- Date of extraction
- Study ID
- Review author ID
- Citation
- Author details
- Abstract/ description
- Aim/objective of study

Methods

- Study design
- Total study duration
- Allocation sequence concealment
- Blinding
- Handling of dropouts/attrition
- Intention to treat analysis?
- Recruitment strategy

Participants

- Total number
- Inclusion criteria
- Number included
- Number excluded
- Drop-outs
- Length of follow-up
- Age
- Sex
- Co-morbidity
- Socio-demographics
- Level of education
- Groups similar at baseline?

Intervention

- Number of intervention groups
- Description of intervention
- Description of control
- Duration of intervention
- Specified: group/individual, pure/blended, caregiver, education, self-management, goal-setting, peer-to-peer contact

Outcomes

- Of each outcome: (together in 1 cell)

1. Baseline mean + sd
2. After intervention + time point (mean+sd)
3. Mean difference + sd
4. Effect size estimate
5. Sample size

- Surrogate endpoints (blood pressure control, glucose control (HbA1c), cholesterol, BMI, physical activity and smoking)
- Clinical outcomes (new cardiovascular events or cardiovascular disease, disability and mortality)
- Psychological outcomes (including depression)
- Behavioural outcomes
- Adherence to drugs
- Quality of life etc (patient satisfaction)
- Use of internet-intervention and adherence

Results

- Number of participants in each group
- Sample size for each outcome
- Drop out and lost to follow-up
- Outcome data for each intervention group
- Estimate of effect with confidence interval
- Adverse events

Conclusions / discussion

- Key conclusions / discussion points & limitations of study authors
- Conclusion / discussion points of review author

Miscellaneous

- Funding source
- Conflict of interest reported
- References to other relevant studies
- Correspondence required?
- Extra comments by review authors

Quality-criteria / risk of bias

- Selection bias
- Performance bias
- Attrition bias
- Detection bias
- Reporting bias
