# Supplementary material for: Web-Based Interventions Targeting Cardiovascular Risk Factors in Middle-Aged and Older People: A Systematic Review and Meta-Analysis
Source: J Med Internet Res. 2016 Mar 11;18(3):e55. doi: 10.2196/jmir.5218 (PMC4808240; doi:10.2196/jmir.5218)
Supplement: Multimedia Appendix 4 [file jmir_v18i3e55_app4.pdf]

Multimedia appendix 4: Summary of risk of bias assessment

Study	1	2	3	4	5	6
Activate your heart 2014	?	+	+	−	+	+
Active after 55 2013	?	?	?	−	−	+
Appel 2011	+	+	+	+	?	+
Avdal 2011	+	?	?	?	+	+
Bennett 2010	+	+	+	+	?	+
Bennett 2012	+	+	+	−	+	+
Bloch 2006	?	−	−	?	?	+
Bond 2007	?	+	+	+	+	+
Bove 2011	−	+	+	−	?	+
Bove 2013	+	+	+	−	?	+
Cho 2006	+	−	−	−	+	+
Diabetes in Check 2014	+	+	+	−	?	+
D-net 2001	+	+	+	−	+	+
e-BP 2008	−	+	+	?	+	+
eCare 2014	+	+	?	−	+	+
E-LITE 2013	?	?	?	+	?	?
EMPOWER-D 2013	?	+	+	+	+	+
Ferney 2009	+	+	+	?	+	+
Grant 2008	+	+	+	−	?	−
Greene 2012	+	?	?	−	−	−
HEART 2014	+	+	+	+	+	+
Heartcare II 2011	−	+	+	−	−	−
Holbrook 2009	+	+	+	+	+	?
Hughes 2011	+	+	+	−	+	+
Ideatel 2000-2010	+	+	+	+	+	+
Keyserling 2014	+	+	+	−	+	+
Kraschnewski 2011	−	?	+	−	?	+
Kwon 2004	?	−	−	+	?	+
Lindsay 2008	+	−	−	−	+	+
Live well 2013	+	+	+	−	+	+
Lorig 2010	−	+	+	−	?	+
Madsen 2008	+	+	+	+	+	+
Magid 2013	+	+	+	+	+	+
McKinstry 2013	+	+	+	+	+	+
McMahon 2012	−	+	+	−	+	+
Mobile DM 2011	?	+	+	?	?	?
My Care Team 2005	−	+	+	−	?	+
My path 2010	+	+	+	?	−	?
Nolan 2012	?	+	+	+	?	?
Peels 2013	?	?	?	−	?	?
Philips Direct Life 2013	+	+	+	−	+	+
POWER 2014	+	+	+	−	−	?
Ralston 2009	−	+	+	−	+	+
REDEEM 2013	+	+	+	−	?	+
Reid 2012	?	+	+	+	+	+
Richardson 2010	−	+	+	−	?	+
Ross 2004	?	+	+	−	+	+
Southard 2003	?	+	+	−	+	?
Stop regain 2008	−	+	?	?	?	+
Suboc 2014	+	?	?	+	+	+
Thiboutot 2013	+	+	+	−	?	+
Verheijden 2004	?	+	+	−	+	+
Vernooij 2012	−	+	+	+	−	+
Webber 2008	?	−	−	+	+	+
Winett 2007	−	+	?	−	+	+
WLM 2008	?	+	+	+	+	+
Zullig 2014	−	+	+	−	?	?

1 Representative sample (selection bias)
2 Random sequence generation (selection bias)
3 Allocation concealment (selection bias)
4 Blinding of outcome assessors (detection bias)
5 Incomplete outcome data (attrition bias)
6 Selective reporting (reporting bias)

+ Low risk of bias
? Unknown risk of bias
− High risk of bias
