# Supplementary material for: Web-Based Interventions Targeting Cardiovascular Risk Factors in Middle-Aged and Older People: A Systematic Review and Meta-Analysis
Source: J Med Internet Res. 2016 Mar 11;18(3):e55. doi: 10.2196/jmir.5218 (PMC4808240; doi:10.2196/jmir.5218)
Supplement: Multimedia Appendix 5 [file jmir_v18i3e55_app5.pdf]

Multimedia appendix 5: Funnel plots

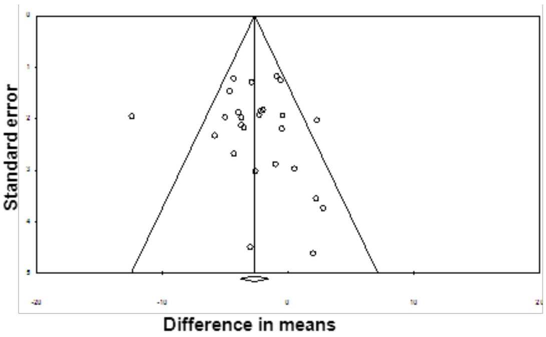

S2.1: systolic BP

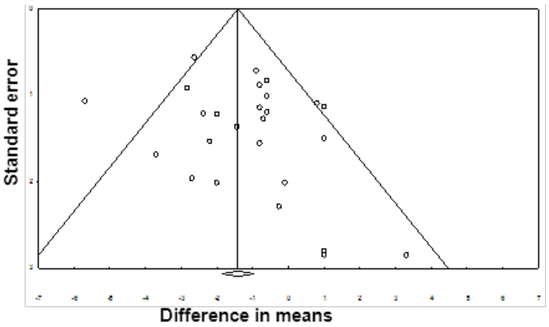

S2.2: diastolic BP

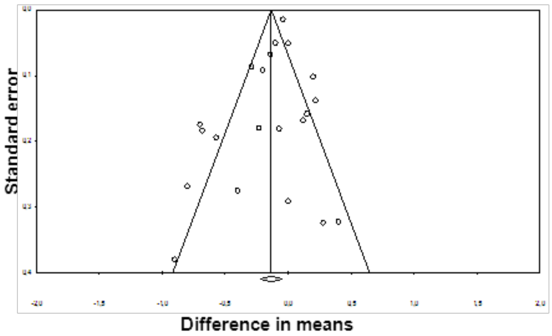

S2.3: HbA1C

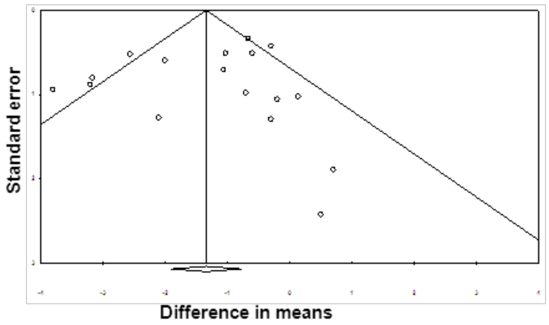

S2.4: weight

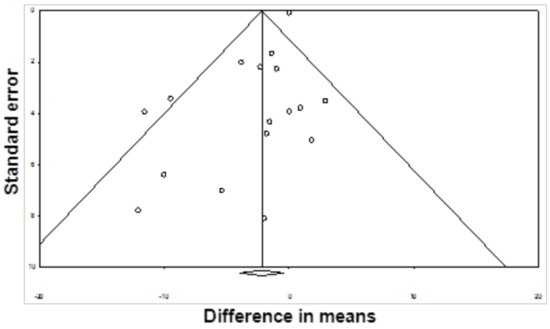

S2.5: LDL-cholesterol

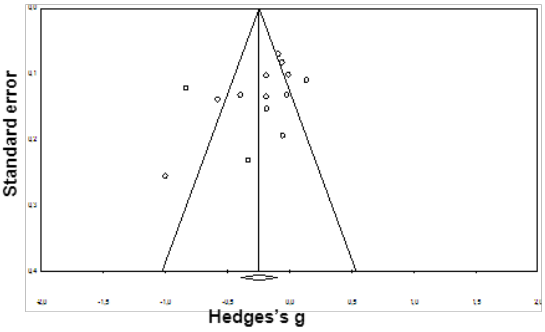

S2.6: physical activity

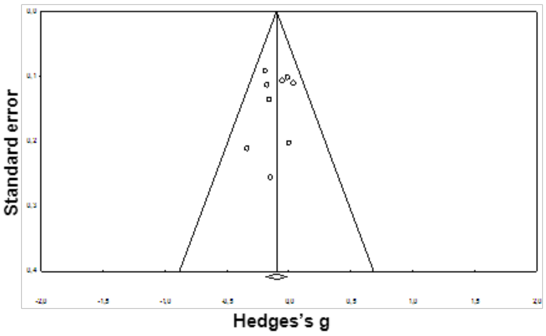

S2.7 cardiovascular composite scores

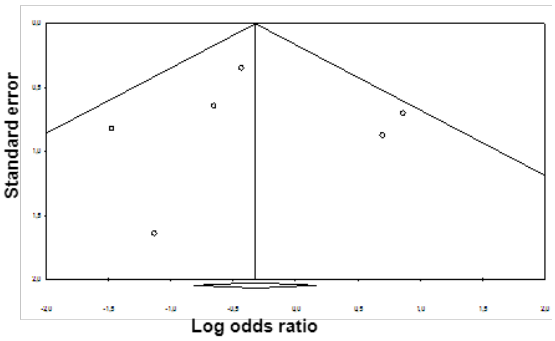

S2.8: new cardiovascular events

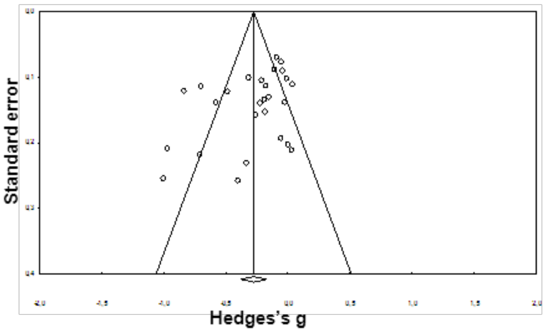

S2.9: pooled primary outcomes
