# Supplementary material for: Web-Based Interventions Targeting Cardiovascular Risk Factors in Middle-Aged and Older People: A Systematic Review and Meta-Analysis
Source: J Med Internet Res. 2016 Mar 11;18(3):e55. doi: 10.2196/jmir.5218 (PMC4808240; doi:10.2196/jmir.5218)
Supplement: Multimedia Appendix 6 [file jmir_v18i3e55_app6.pdf]

Multimedia appendix 6: Subgroup analysis: study-duration

Outcome	N of studies	Effect size	Lower 95%CI	Upper 95%CI	I^2
**Systolic BP (mmHg)**					
short (<12 months)	12	-3.54	-5.66	-1.41	63%
long (≥12 months)	14	-2.01	-3.12	-0.90	25%
**Diastolic BP (mmHg)**					
short (<12 months)	12	-1.82	-3.11	-0.54	60%
long (≥12 months)	14	-0.99	-1.62	-0.36	17%
**HbA1c (%)**					
short (<12 months)	8	-0.23	-0.36	-0.10	82%
long (≥12 months)	13	-0.06	-0.21	0.08	69%
**Weight (kg)**					
short (<12 months)	7	-1.86	-2.17	-1.00	70%
long (≥12 months)	10	-0.84	-1.53	-0.14	41%
**LDL-cholesterol (mg/dl)**					
short (<12 months)	5	-0.32	-1.33	0.69	9%
long (≥12 months)	12	-2.98	-5.60	-0.36	32%
**Physical activity (Hedges' *g*)**					
short (<12 months)	8	-0.37	-0.62	-0.11	86%
long (≥12 months)	6	-0.12	-0.25	0.01	56%
**CV composite scores (Hedges' *g*)**					
short (<12 months)	4	-0.18	-0.31	-0.05	0%
long (≥12 months)	5	-0.05	-0.16	0.05	0%
**Primary outcomes (Hedges' *g*)**					
short (<12 months)	15	-0.43	-0.57	-0.29	69%
long (≥12 months)	22	-0.12	-0.19	-0.06	41%
