# Supplementary material for: Web-Based Interventions Targeting Cardiovascular Risk Factors in Middle-Aged and Older People: A Systematic Review and Meta-Analysis
Source: J Med Internet Res. 2016 Mar 11;18(3):e55. doi: 10.2196/jmir.5218 (PMC4808240; doi:10.2196/jmir.5218)
Supplement: Multimedia Appendix 7 [file jmir_v18i3e55_app7.pdf]

Multimedia appendix 7: Risk of bias assessment: sensitivity analyses for the domains of risk of bias

Domain	N of studies	Hedges' *g*	Lower 95%CI	Upper 95%CI limit
**Representative sample**				
Low risk	25	-0.25	-0.35	-0.16
Unclear / high risk	22	-0,26	-0,36	-0,15
**Random sequence generation**				
Low risk	39	-0,20	-0,27	-0,14
Unclear / high risk	8	-0,53	-0,82	-0,24
**Allocation concealment**				
Low risk	37	-0,22	-0,29	-0,15
Unclear / high risk	10	-0,38	-0,60	-0,17
**Blinding of outcome assesment**				
Low risk	15	-0,37	-0,50	-0,23
Unclear / high risk	32	-0,20	-0,28	-0,12
**Incomplete outcome data**				
Low risk	25	-0,26	-0,35	-0,17
Unclear / high risk	22	-0,25	-0,36	-0,14
**Selective reporting**				
Low risk	38	-0,28	-0,36	-0,20
Unclear / high risk	9	-0,12	-0,23	-0,00
